# Dendrimers Improve Apolipoprotein Nanoparticle mRNA Delivery to Immune Cells

**DOI:** 10.1002/adma.202504830

**Published:** 2025-09-12

**Authors:** Mirre M. Trines, Daniek Hoorn, Stijn R.J. Hofstraat, Robby C. Zwolsman, Tom Anbergen, Iris Versteeg, Yuri van Elsas, Jeroen Deckers, Merel M.A. Hendrikx, Teun Kleuskens, Youssef B. Darwish, Gijs W.B. Ros, Sjoerd F. Dijkstra, Bram Priem, Matt Timmers, P. Michel Fransen, Maarten J. Pouderoijen, Bas F.M. de Waal, E.W. Meijer, Thijs J. Beldman, Yohana C. Toner, Ewelina Kluza, Willem J.M. Mulder, Henk M. Janssen, Roy van der Meel

**Affiliations:** ^1^ Laboratory of Chemical Biology Department of Biomedical Engineering and Institute for Complex Molecular Systems (ICMS) Eindhoven University of Technology Eindhoven 5600 MB The Netherlands; ^2^ Department of Internal Medicine and Radboud Center for Infectious Diseases (RCI) Radboud University Medical Center Nijmegen 6525 GA The Netherlands; ^3^ Biotrip B.V. Eindhoven 5641 AM The Netherlands; ^4^ SyMO‐Chem B.V. Eindhoven 5612 AZ The Netherlands; ^5^ Laboratory of Macromolecular and Organic Chemistry Institute for Complex Molecular Systems (ICMS) Eindhoven University of Technology Eindhoven 5600 MB The Netherlands

**Keywords:** apolipoprotein nanoparticles, dendrimers, ionizable cationic lipids, mRNA delivery

## Abstract

Employing messenger RNA (mRNA) for protein production in the liver or for vaccine purposes is a promising therapeutic approach. However, unlocking mRNA's full therapeutic potential requires systemic delivery platform technology with controllable biodistribution features. Apolipoprotein nanoparticles (aNP) containing monovalent ionizable cationic lipids have been shown to functionally deliver mRNA to myeloid progenitor cells in the bone marrow after intravenous administration. Here, the development of polyvalent ionizable cationic dendrimers is reported for incorporation in aNPs to enable efficient mRNA complexation and functional delivery. A library of dendrimers is first rationally designed with diverse hydrophobic core units, a number of branching units, and functionalized terminal units. Upon incorporation, eleven distinct dendrimer‐based aNP‐mRNA formulations are screened and characterized in vitro for their properties. Based on the screening outcome, four formulations are selected and evaluated their ability to induce functional gene expression in vivo. The results indicate that the lead polyvalent dendrimer‐based aNP formulation outperformed formulations containing a clinically approved ionizable cationic lipid regarding gene expression in hematopoietic stem and progenitor cells in the bone marrow after intravenous administration.

## Graphical abstract

Apolipoprotein nanoparticle (aNP) platform technology efficiently delivers RNA to immune cells following systemic administration. Here, it is demonstrated that incorporating polyvalent ionizable cationic dendrimers in aNPs enables robust mRNA complexation and functional delivery. Dendrimer‐based aNP‐mRNA formulations outperform formulations containing a clinically approved ionizable cationic lipid regarding gene expression in hematopoietic stem and progenitor cells in the bone marrow after intravenous administration.

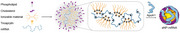

## 1 Introduction

The ability to provide cells with a blueprint for producing therapeutic proteins by introducing exogenous messenger RNA (mRNA) is revolutionizing healthcare. However, using mRNA therapeutically is dependent on chemical modifications that reduce immunostimulatory effects^[^ ^1^ ^]^ and sophisticated nanotechnology that prevents degradation and ensures intracellular delivery of the nucleic acid payload.^[^ ^2^ ^]^ Over the course of the last decades, lipid nanoparticle (LNP) technology was developed that proved to be crucial for the clinical translation of the first small interfering RNA (siRNA) drug,^[^ ^3^, ^4^ ^]^ the COVID‐19 mRNA vaccines,^[^ ^5^, ^6^ ^]^ and gene editing approaches.^[^ ^7^, ^8^, ^9^ ^]^ Nevertheless, current clinically relevant LNP systems are mostly suited for vaccine purposes following local administration,^[^ ^10^ ^]^ or for hepatic delivery following systemic administration, owing to the LNPs’ liver tropism.^[^ ^11^, ^12^ ^]^

To overcome LNP's inherent propensity for hepatic accumulation, compositional and antibody surface modifications are being explored. For example, incorporating modified ionizable cationic materials or permanently charged lipids in LNPs has been shown to skew their biodistribution toward the spleen, lungs, or other tissues^[^ ^13^, ^14^, ^15^, ^16^ ^]^ while LNP library screening has identified formulations that efficiently deliver mRNA to distinct cell types.^[^ ^17^, ^18^ ^]^ Moreover, conjugating antibodies to LNPs has been used to promote interactions with leukocytes,^[^ ^19^ ^]^ including T cells^[^ ^20^, ^21^, ^22^ ^]^ and to hematopoietic stem and progenitor cells (HSPCs).^[^ ^23^, ^24^ ^]^

We recently introduced the apolipoprotein nanoparticle (aNP) platform technology specifically designed to deliver RNA to immune cells.^[^ ^25^ ^]^ By integrating high‐density lipoprotein (HDL)’s main component, apolipoprotein A1 (apoA1),^[^ ^26^ ^]^ the aNP platform leverages HDL's capacity to interact with myeloid cells^[^ ^27^ ^]^ and ability to transport RNA.^[^ ^28^ ^]^ In our previous study, we showed that aNPs containing established monovalent ionizable cationic lipids, including DLin‐MC3‐DMA^[^ ^29^, ^30^ ^]^ and ALC‐0315, efficiently delivered siRNA and mRNA, respectively, to myeloid cells and their progenitors in the bone marrow.^[^ ^25^ ^]^

Here, we report the development of polyvalent ionizable cationic dendrimers for efficient mRNA complexation and incorporation in aNPs. After establishing a suitable composition that stably incorporated mRNA, we screened and characterized the properties of eleven distinct dendrimer‐based aNP‐mRNA formulations in vitro. From this small library, we selected four formulations and evaluated their ability to induce functional mCherry reporter gene expression in vivo. We demonstrate that the lead polyvalent dendrimer‐based aNP formulation outperformed aNPs and LNPs containing ALC‐0315 regarding gene expression in HSPCs in the bone marrow.

## 2 Results

### 2.1 Designing Polyvalent Ionizable Cationic Dendrimers for aNP Incorporation

**Figure 1:**
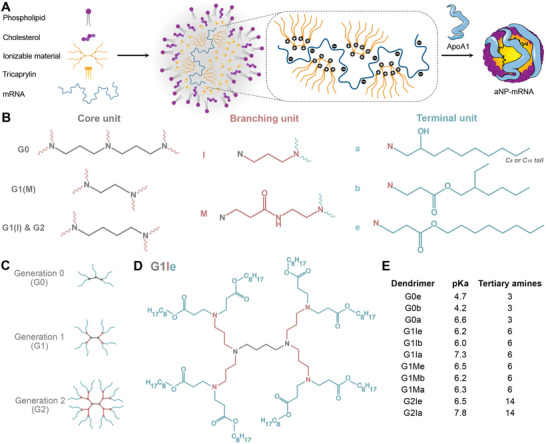
Polyvalent ionizable cationic dendrimers for mRNA complexation and incorporation in aNPs. A) Schematic representation of multicomponent apolipoprotein A1 (apoA1)‐based lipid nanoparticles containing mRNA (aNP‐mRNA). B) Overview of dendrimer structural components consisting of a core unit, a branching unit, and a terminal unit. C) Dendrimer generations are determined by the number of branching units spacing the core and terminal units. D) Example structure of lead dendrimer G1Ie, based on generation 1 (G1), with PPI branching unit (I) and ester tail units (e). E) Dendrimer identifiers, pKa values, and number of tertiary amines.

To generate aNP‐mRNA formulation, we have previously established a two‐step flow manufacturing process using a T‐junction mixer.^[^ ^25^, ^31^ ^]^ By rapidly mixing lipid components, including ionizable cationic materials in an organic phase with mRNA in buffer at low pH, supramolecular lipid nanoparticles self‐assemble based on charge and hydrophobic interactions. The subsequent addition of apoA1 results in stable aNPs with efficient mRNA incorporation (**Figure** **1**A). This procedure was used to produce a prototype aNP‐mRNA containing the ionizable cationic lipid ALC‐0315 used for the COVID‐19 mRNA vaccine formulation tozinameran.^[^ ^5^ ^]^ Compared to an LNP‐mRNA control formulation, the aNP‐mRNA formulation induced significantly more reporter gene expression in bone marrow progenitors after intravenous administration in mice.^[^ ^25^ ^]^

ALC‐0315 is a monovalent ionizable amino lipid with a reported apparent pKa of 6.09.^[^ ^32^ ^]^ During aNP or LNP production at low pH (≤4), the lipid's tertiary amine becomes protonated, yielding an ammonium cation that interacts with the mRNA's negatively charged phosphate groups. At physiological pH, ALC‐0315 ensures a net neutral nanoparticle surface charge, while its protonation in the endosome following cell uptake promotes endosomal escape.^[^ ^33^ ^]^ Besides monovalent ionizable amino lipids, polyvalent ionizable materials, including lipids,^[^ ^34^ ^]^ lipidoids,^[^ ^35^, ^36^ ^]^ and dendrimers^[^ ^37^, ^38^ ^]^ have been developed and incorporated in LNPs for efficient nucleic acid complexation. Given the increased number of tertiary amines per molecule compared to monovalent molecules, dendrimers’ higher charge density and well‐defined architecture provide potential benefits for mRNA complexation and delivery.^[^ ^39^, ^40^ ^]^ In the past, we introduced dendrimers based on poly(propyleneimine) (PPI) with a large variety of modifications.^[^ ^41^, ^42^ ^]^ The PPI structure can give the highest concentration of protonated amines as used for DNAzymes delivery in cells,^[^ ^39^ ^]^ and for phosphate binding in Bixalomer.^[^ ^43^ ^]^

We therefore set out to develop polyvalent ionizable amino dendrimers for efficient mRNA complexation and stable incorporation in the aNP platform. To this end, we first rationally designed polyvalent ionizable dendrimers composed of a hydrophobic core unit and terminal units that are spaced by one or more branching units (Figure 1B). The generation number of a dendrimer is determined by the number of branching units spacing the core and terminal units (Figure 1C). The molecules are synthesized through a repetitive sequence of reactions, with each repetition yielding a higher dendrimer generation with twice the number of end groups and approximately double the molecular weight.^[^ ^44^ ^]^ While generation 0 (G0) dendrimers are composed of a bis(3‐aminopropyl)amine core directly connected to terminal units, generation 1 (G1) dendrimers are either classified as PPI or poly(amidoamine) (PAMAM) with a 1,4‐diaminobutane core, and generation 2 (G2) materials only include PPI branching units.^[^ ^45^ ^]^ From the inner core unit, the dendrimers branch symmetrically toward the outer terminal units, which are either linear or branched aliphatic alkyl chains. These terminal units are functionalized with a hydroxyl or an ester group, enhancing biodegradability and thereby reducing cytotoxicity,^[^ ^39^ ^]^ resulting in a final dendrimer structure such as G1Ie (Figure 1D).

After synthesizing a small library of 11 diverse polyvalent dendrimers (see Dendrimer synthesis, Supporting Information), we determined their pKa values using a 6‐p‐toluidino‐2‐naphthalenesulfonic acid (TNS) assay (Figure 1E; Figure S1, Supporting Information). While G0e and G0b dendrimers had low pKa values of 4.7 and 4.2, respectively, one dendrimer (G2Ia) had a relatively high pKa value of 7.8. In contrast, several G1 dendrimers (G1Ie, G1Ib, G1Me, G1Mb, G1Ma) had pKa values between 6.0 and 6.5, which has been reported as favorable for endosomal escape of RNA therapeutics.^[^ ^30^, ^46^, ^47^ ^]^ Following the successful synthesis and analysis of the polyvalent dendrimers, we next investigated their suitability for aNP incorporation.

### 2.2 Establishing a Dendrimer aNP‐mRNA Composition

**Figure 2:**
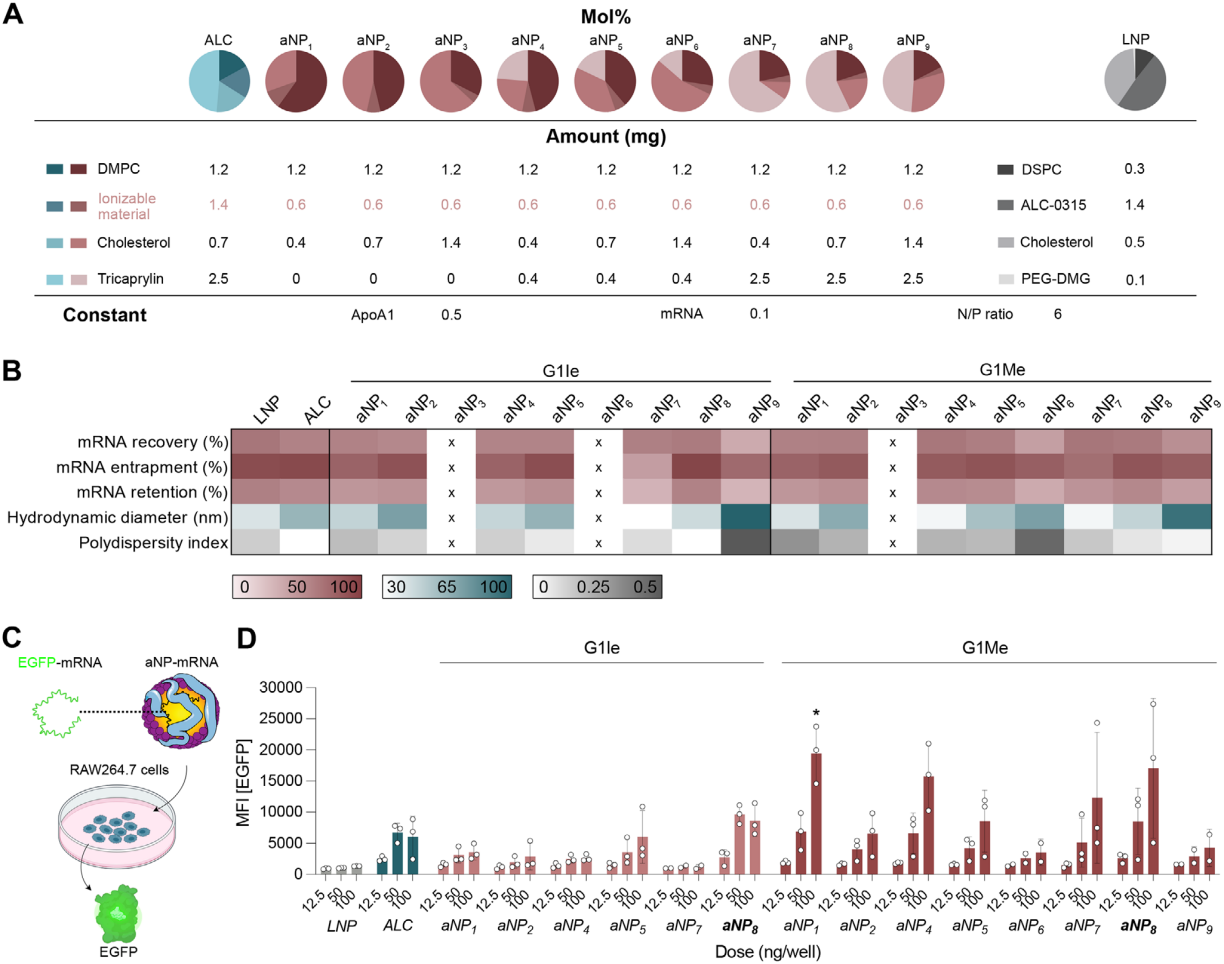
Establishing optimal aNP compositions containing dendrimers. A) Nine selected aNP compositions, an aNP with ALC‐0315 (ALC), and an LNP presented in molar percentages and in milligrams. B) Physicochemical analysis of aNPs and control LNP. Compositions indicated by an x did not form stable aNPs. C) Schematic showing in vitro transfection of RAW264.7 macrophages with aNP‐mRNA EGFP. D) Mean fluorescence intensity (MFI) of expressed EGFP protein 24 h after in vitro transfection of RAW264.7 cells with different nanoparticle compositions, obtained with flow cytometry. Data represent mean ± SD (n = 3 experiments) and were analyzed using a one‐way ANOVA with Dunnett's post hoc test. Significant differences between ALC‐based aNP (100 ng) versus ionizable material‐based aNP or LNP (100 ng) are indicated by: ^*^ indicates statistical difference (*p* ≤0.05).

To identify a suitable aNP composition, we selected two structurally diverse G1 dendrimers containing six tertiary amines and a PPI (G1Ie) or PAMAM (G1Me) branching unit. Using our rapid mixing procedure, we incorporated the dendrimers in nine distinct formulations based on a prototype aNP‐mRNA composition we previously established.^[^ ^25^ ^]^ While varying the ratios between 1,2‐dimyristoyl‐sn‐glycero‐3‐phosphocholine (DMPC) to cholesterol and triglycerides (TG), we maintained constant amounts of DMPC, apoA1, and mRNA. We also kept a constant nitrogen‐over‐phosphate (N/P) ratio of 6, similar to the COVID‐19 mRNA vaccines^[^ ^48^ ^]^ (**Figure** **2**A; Table S1, Supporting Information).

Incorporating G1Ie in the aNP_3_ and aNP_6_ compositions did not yield stable formulations (Figure 2B). The same held true for G1Me in aNP_3_, which may be due to the composition's high cholesterol content (>50 mol%). In addition, although the high cholesterol content of aNP_9_ composition produced stable formulations, its physicochemical properties were unfavorable, with poor mRNA encapsulation and dispersity. Most stable formulations had mRNA entrapment values higher than 70%, with compositions aNP_5_ and aNP_8_ having the highest entrapment values above 80%. Notably, although the mol% of ionizable cationic dendrimers used for aNP_5_ (6.2 mol%) and aNP_8_ (3.2 mol%) was substantially less than aNP (17 mol%) and LNP (50 mol%) formulations containing the monovalent ionizable cationic lipid ALC‐0315, mRNA entrapment efficiencies were similar (Tables S1 and S2, Supporting Information). Increasing the cholesterol content impacted aNPs’ hydrodynamic diameter, with particle sizes ranging from 60 to 100 nm in diameter, as determined by dynamic light scattering (DLS). Despite this variation, formulations were homogenous, with a polydispersity index below 0.25 for almost all aNP compositions (Table S2, Supporting Information).

To determine the aNPs’ in vitro functionality, we treated murine RAW264.7 macrophages with aNPs containing enhanced green fluorescent protein (EGFP) mRNA at three increasing doses (Figure 2C). Flow cytometry analysis revealed dose‐dependent expression for all aNP compositions, indicated by the percentage EGFP‐positive cells (Figure S2, Supporting Information) and mean fluorescence intensity (Figure 2D). We found composition aNP_8_ to have favorable physicochemical properties and the ability to induce considerable reporter gene expression when formulated using both G1Ie and G1Me. In addition, these aNP formulations induced higher EGFP expression when compared to control aNPs based on ALC‐0315. Since composition aNP_8_ was suitable for both dendrimers, we selected this composition for further dendrimer screening.

### 2.3 Characterizing and Screening Dendrimer aNP‐mRNA Formulations

**Figure 3:**
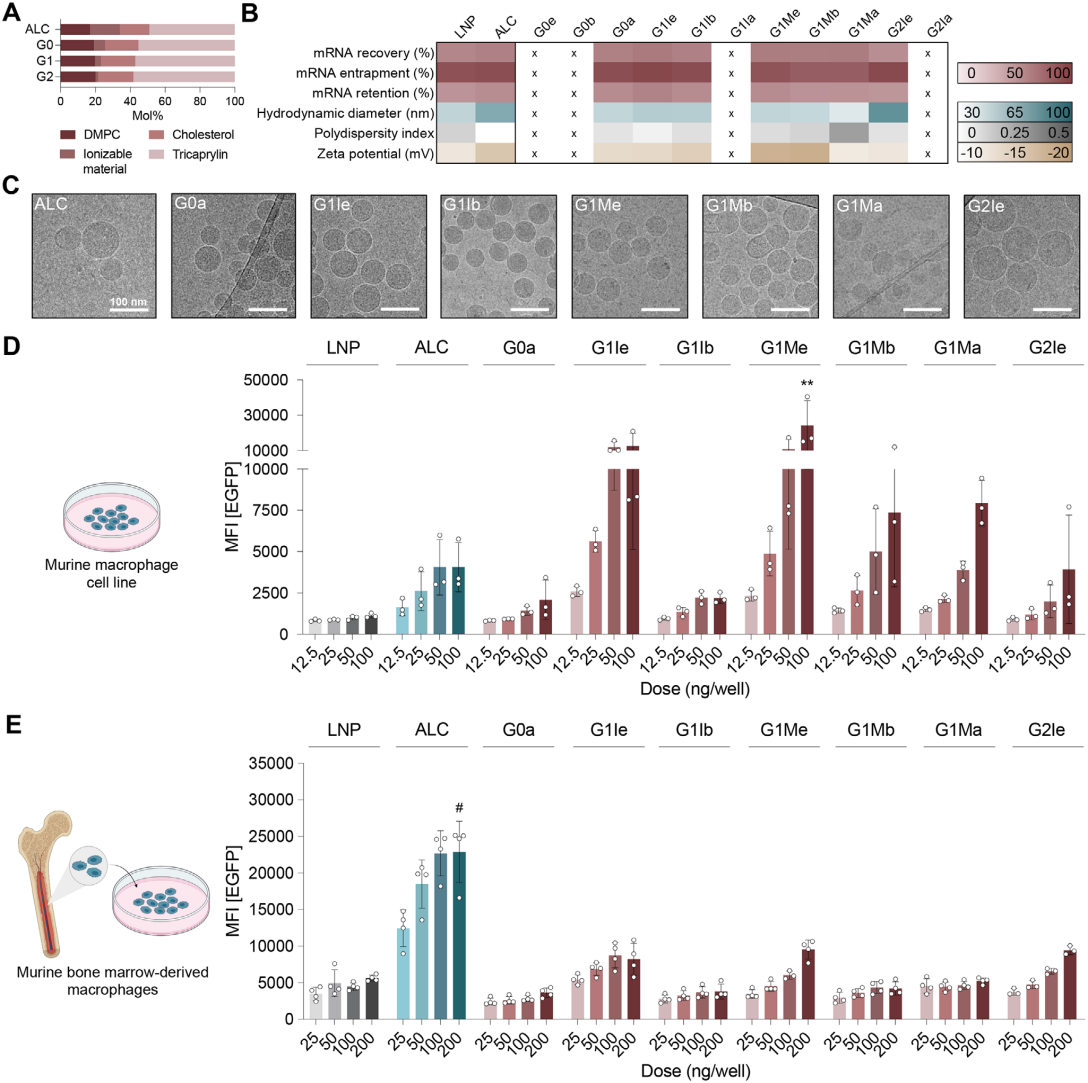
Characterization and in vitro evaluation of dendrimer aNP‐mRNA formulations. A) aNP compositions in mol percentages. B) Physicochemical analysis of aNPs and control LNP. Compositions indicated by an x did not form stable aNPs. C) Cryogenic transmission electron microscopy (cryo‐TEM) analysis of dendrimer aNP formulations. Scale bar represents 100 nm. D) EGFP mean fluorescent intensity (MFI) after aNP‐mRNA treatment in RAW264.7 macrophages, and E) murine bone marrow‐derived macrophages. Data represent mean ± SD (n = 3‐4 experiments) and were analyzed using a one‐way ANOVA with Dunnett's post hoc test. Significant differences between ALC‐based aNP‐mRNA (100 or 200 ng) versus dendrimer‐based aNP‐mRNA or LNP‐mRNA (100 or 200 ng) are indicated by: ^*^ indicates statistical difference (*p* ≤ 0.05), ^**^ indicates statistical difference (*p* ≤ 0.01), # indicates statistical difference (*p* ≤ 0.0001) from all other groups.

Using the optimized composition aNP_8,_ we formulated aNPs containing the eleven distinct dendrimers and compared their properties with aNP and LNP formulations based on ALC‐0315. As previously established, increasing the dendrimer generation and subsequent number of tertiary amines significantly reduced the required molar percentage of ionizable material (**Figure** **3**A and Table S1, Supporting Information). For aNPs based on ALC‐0315, 1.41 mg was used (17 mol%), while this was less for aNPs containing dendrimers of G0 (0.64 mg, 6.4 mol%), G1 (0.55 mg, 3.3 mol%), and G2 (0.49 mg, 1.5 mol%). As expected, dendrimers with pKa values <5.0 (G0e and G0b) and >7.0 (G1Ia and G2Ia) did not yield stable formulations when formulated at pH 4, while all other dendrimers resulted in stable aNPs with mRNA entrapment values >80%, which was comparable to control aNP and LNP formulations containing ALC‐0315 (Figure 3B; Table S3, Supporting Information). Except for G2Ie aNPs having a hydrodynamic diameter of ≈80 nm, all dendrimer‐based aNPs had sizes ≈50 nm and were smaller than ALC‐0315 aNPs. Nearly all aNPs exhibited a favorable polydispersity index below 0.15 and a negative zeta potential ≈−15 mV. To confirm aNP size distribution, homogeneity, and determine morphology, we analyzed the dendrimer aNP formulations using cryogenic transmission electron microscopy (cryo‐TEM) (Figure 3C; Figure S3, Supporting Information). While all dendrimer aNP formulations exhibited spherical morphology, several differences were observed between formulations. For example, G2Ie aNPs displayed slightly larger particles with lamellar membrane structures, while G1Mb‐aNPs showed multicompartmental blebs within the inner particle structure.^[^ ^49^, ^50^ ^]^

Following the individual dendrimer aNP formulations’ physicochemical analysis, we determined their ability to induce reporter EGFP expression in RAW264.7 macrophages and murine bone marrow‐derived macrophages (BMDMs). Following treatment with four increasing aNP‐mRNA doses, the percentage of EGFP‐positive cells (Figure S4A,B, Supporting Information) and expression levels (Figure 3D,E) were determined by flow cytometry. All aNPs induced dose‐dependent EGFP expression in the macrophage cell line and primary cells. Furthermore, we observed that G1Me aNPs induced significantly higher EGFP expression in RAW264.7 macrophages compared to control formulations and other dendrimer aNPs (Figure 3D). G1Ie and G1Me aNPs also induced considerable EGFP expression in BMDMs (Figure 3E). Notably, ALC‐0315 aNPs exhibited a significantly higher EGFP signal in BMDMs compared to RAW264.7 macrophages. In addition, no changes in cell viability were observed in both cell types following aNP exposure (Figure S4A,B, Supporting Information). Based on these findings, we selected the two well‐performing (G1Ie and G1Me) and two less‐performing (G1Ib and G1Mb) dendrimer aNPs for in vivo evaluation.

### 2.4 Evaluating Dendrimer aNPs’ Capacity for mRNA Delivery to Immune Cells In Vivo

**Figure 4:**
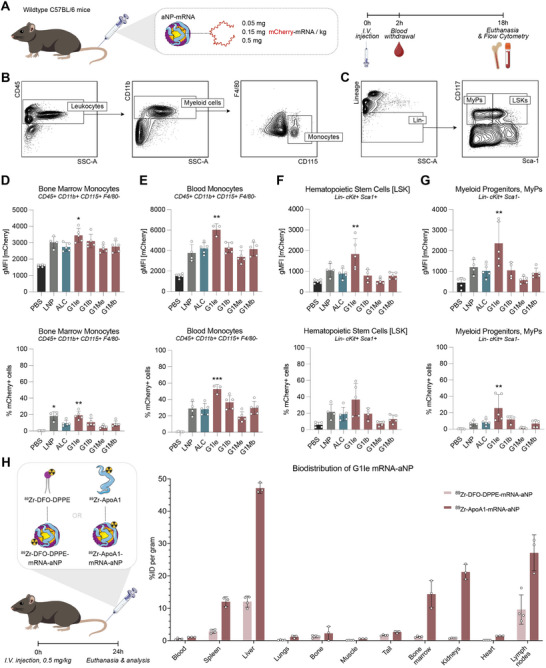
Evaluation of dendrimer‐based aNPs for mRNA delivery to immune cells in mice. A) Schematic workflow showing mice receiving an intravenous (i.v.) injection of mCherry‐encoding aNP‐mRNA to C57BL/6J mice at doses of 0.05, 0.15, or 0.5 mg kg^−1^ and blood withdrawal for serum interleukin‐6 levels. mCherry expression levels were determined in bone marrow and blood 18 h after injection using flow cytometry. Flow cytometry gating strategy for B) myeloid cells and C) progenitor cells. An extended overview of the gating strategy can be found in Figure S6 (Supporting Information). D–G) mCherry expression in D) bone marrow monocytes, E) blood monocytes, and bone marrow stem and progenitor cells indicated by geometric mean fluorescence intensity (gMFI) and percentage (%) of mCherry positive cells 18 h following i.v. injection of LNP or aNP containing mCherry mRNA at a dose of 0.15 mg kg^−1^. Data represent mean ± SD (n = 3–5 mice) and was analyzed using a one‐way ANOVA with Dunnett's post hoc test. Significant differences between ALC‐based aNP‐mRNA versus dendrimer‐based aNP‐mRNA or LNP‐mRNA are indicated by: ^*^ indicates statistical difference (*p* ≤ 0.05), ^**^ indicates statistical difference (*p* ≤ 0.01), ^***^ indicates statistical difference (*p* ≤ 0.001). H) Quantitative biodistribution of G1Ie aNP‐mRNA 24 h after intravenous administration in mice as determined by ex vivo gamma counting. aNPs were either lipid labeled (^89^Zr‐DFO‐DPPE) or protein labeled (^89^Zr‐apoA1). Data represent mean ± SD (n = 3‐4 mice).

To determine dendrimer aNPs’ ability to deliver functional mRNA to immune cells and HSPCs in the bone marrow, mRNA encoding the fluorescent reporter protein mCherry was formulated in aNP_8_ using the four selected dendrimers G1Ie, G1Me, G1Ib, and G1Mb (Figure S5, Supporting Information). The physicochemical properties of the dendrimer aNPs were similar to the formulations used for in vitro studies (Table S4, Supporting Information). Next, we intravenously injected the dendrimer aNP‐mRNA to C57BL/6J mice using three different doses (0.05, 0.15, or 0.5 mg kg^−1^ mRNA) alongside an LNP‐mRNA (0.15 mg kg^−1^ mRNA) and phosphate buffered saline (PBS) control, and determined mCherry expression using flow cytometry (**Figure** **4**A). 18 h after aNP administration, we sacrificed the mice and collected whole blood and bone marrow cells. The cells were subsequently stained using an antibody panel, optimized for the detection of bone marrow stem cells, progenitor cells, and mature myeloid cells using flow cytometry (Figure 4B,C; Figure S6, Tables S5 and S6, Supporting Information).

Our data reveal significantly more mCherry‐positive cells and higher expression levels in mice injected with G1Ie aNP‐mRNA compared to ALC‐0315‐based formulations in mature myeloid cells in the bone marrow and blood at a dose of 0.15 mg kg^−1^ (Figure 4D,E; Figure S7A,B, Supporting information). Importantly, G1Ie‐based aNP‐mRNA also induced significantly higher reporter gene expression in hematopoietic stem cells and myeloid progenitor cells compared to ALC‐0315‐based aNP and LNP controls (Figure 4F,G; Figure S7C,D, Supporting information), highlighting the potential of these polyvalent dendrimer‐based aNPs to efficiently deliver mRNA to immune cells.

Besides functional effects, we collected blood samples 2 h after aNP‐mRNA administration and determined interleukin‐6 (IL‐6) and tumor necrosis factor (TNF) cytokine levels in serum using ELISA (Figure S8A,B, Supporting Information). In line with increased mCherry expression levels, we observed elevated IL‐6 and TNF serum levels for G1Ie aNPs compared to ALC‐0315‐based aNPs and LNPs. At 24 h after administration, we found no significant differences in serum liver enzyme levels and kidney toxicity markers of mice treated with dendrimer aNP‐mRNA formulations compared to aNP and LNP controls (Figure S8C, Supporting Information). In addition, we observed no notable tissue damage using histological analyses of liver sections following aNP‐mRNA treatment (Figure S8D, Supporting Information).

After establishing G1Ie aNP‐mRNA's ability to induce robust reporter gene expression in myeloid cells in the blood and their progenitors in the bone marrow, we assessed this formulation's in vivo behavior following intravenous administration. According to methods previously established by Pérez‐Medina et al.,^[^ ^51^ ^]^ we either radiolabeled G1Ie aNP‐mRNA through apoA1 or phospholipids with zirconium‐89 (^89^Zr) and quantitatively determined biodistribution by ex vivo gamma counting. We first determined that incorporating 1,2‐dipalmitoyl‐sn‐glycero‐3‐phosphoethanolamine (DPPE) or apoA1 functionalized with 89Zr chelator desferrioxamine B (DFO) did not impact G1Ie aNP's physicochemical properties compared to unlabeled formulations (Table S4, Supporting Information). In line with previous reports on radiolabeled apolipoprotein nanoparticles, at 24 h following intravenous administration of ^89^Zr‐radiolabeled G1Ie aNP‐mRNA,^[^ ^51^ ^]^ ex vivo gamma counting revealed distinct biodistribution patterns for apoA1 and the phospholipids, with varying accumulation in the liver, spleen, bone marrow, and lymph nodes (Figure 4H). We have previously shown that multicomponent aNPs containing ^89^Zr‐siRNA have a relatively short blood half‐life (<120 min) and rapidly accumulate in the bone marrow, spleen, and liver following intravenous injection.^[^ ^25^ ^]^

In summary, intravenous administration of G1Ie aNP‐mRNA results in mRNA accumulation in hematopoietic organs and translation in myeloid (progenitor) cells, with the formulation's natural carrier components, i.e., apoA1 and phospholipids, behaving endogenously.

## 3 Conclusion

In this study, we designed a series of polyvalent ionizable cationic dendrimers and successfully incorporated them in aNPs for efficient mRNA complexation. After screening and characterizing a small library of dendrimer aNP formulations in vitro, we evaluated the ability of four formulations to functionally deliver mRNA in vivo. Using flow cytometry analysis of reporter gene expression in bone marrow HSPCs, we identified G1Ie as the lead dendrimer. Compared to aNP and LNP formulations containing the clinically approved monovalent ionizable cationic lipid ALC‐0315, G1Ie‐aNPs functionally delivered mRNA to significantly more myeloid progenitor cells in the bone marrow following intravenous injection, resulting in higher expression levels.

A key factor in unleashing mRNA's full therapeutic potential is controlling its biodistribution, and tissue‐ or even cell‐specific delivery following systemic administration. Although an increasing number of studies demonstrate enriched mRNA localization in the lungs, spleen, and liver using LNPs, reaching therapeutically relevant levels of bone marrow HSPCs remains challenging. Despite compositional modifications^[^ ^18^, ^52^ ^]^ and surface antibody conjugation^[^ ^23^, ^24^ ^]^ of LNPs to achieve this, aNPs were specifically designed for RNA delivery to immune cells and HSPCs^[^ ^25^ ^]^ in contrast to LNPs.

While incorporating the lead dendrimer G1Ie in aNPs resulted in robust functional mRNA delivery, further analysis, optimization, and development of polyvalent ionizable cationic dendrimers is required to enable their use for a wide range of therapeutic applications. Ideally, ionizable cationic materials are designed in such a way that warrants their potency, but also ensures they are immunosilent and biodegradable, resulting in an optimal safety profile.^[^ ^53^, ^54^ ^]^ Recently developed machine learning approaches taking both these features can accelerate the development of these nanomaterials.^[^ ^55^, ^56^, ^57^, ^58^ ^]^

In conclusion, our study demonstrates that the rational design of polyvalent ionizable cationic dendrimers and their subsequent incorporation in aNPs is an attractive approach to improve the platform's functional mRNA delivery to HSPCs in the bone marrow. Given that the modular platform is composed of predominantly natural materials, its potent functional effects and favorable safety profile make it aptly suited for diverse immunotherapeutic applications, including vaccination and immuno‐oncology.

## 4 Experimental Section

### Materials

DMPC, DSPC, and PEG (2K)‐DMG were purchased from Avanti Polar Lipids. Cholesterol, tricaprylin, and sodium acetate were purchased from Sigma. ALC‐0315 was obtained from SyMO‐Chem B.V. TNS was purchased from Abcam.

### Methods—Synthesis of Polyvalent Ionizable Dendrimers

Polyvalent ionizable dendrimer synthesis is described in the Supporting Information.

### Methods—TNS pKa Analysis Assay

A TNS master buffer was prepared containing 150 mm sodium chloride, 10 mM potassium monophosphate, 10 mm sodium borate, and 10 mM citrate. The pH was adjusted to values ranging from 3.5 to 10 in increments of 0.5 by adding 1 m HCl or 1 m NaOH. Polyvalent ionizable dendrimers were, depending on their number of tertiary amines, diluted to a concentration of 166.6, 83.3, or 35.7 µm in ethanol (for dendrimers with 3, 6, or 14 tertiary amines, respectively). 6‐p‐Toluidino‐2‐naphthalenesulfonic acid (TNS) was diluted to a concentration of 10 mm in DMSO. Next, a TNS working solution was prepared by diluting the 10 mm TNS solution in DMSO with milliQ to a concentration of 83 µm. In a black 96‐well plate, 90 µL of buffer solution was added to the wells in triplicate. Subsequently, 5 µL of lipid solutions were added to each well, and, immediately after, 5 µL of TNS working solution was added. The plate was incubated for 20 min at room temperature on an orbital shaker (400 rpm), protected from light. Fluorescence was measured using a Tecan Spark microplate reader at an excitation wavelength of 318 nm and an emission wavelength of 443 nm. A four‐parameter logistic curve was used to calculate the pKa.

### aNP‐mRNA Formulation

mRNA‐loaded aNPs were formulated via rapid T‐junction mixing as described previously.^[^ ^25^, ^31^ ^]^ Briefly, lipids (DMPC, tricaprylin, cholesterol, and ionizable cationic dendrimers or ALC‐0315 for aNPs; DSPC, cholesterol, PEG‐DMG, and ALC‐0315 for LNPs) were dissolved in ethanol in specific ratios (Table S4, Supporting Information) and rapidly mixed with 25 mm sodium acetate (pH 4) containing mRNA. Organic and aqueous phases were mixed at a flow rate ratio of 1:3 (organic/aqueous) and directly collected in a 12–14 kDa MWCO dialysis membrane bag (Spectra/Por). Formulations were dialyzed overnight against 1X PBS (pH 7.4) at 4 °C. The subsequent day, samples were collected from the dialysis bags, and the volume was determined. To generate aNPs, the desired amount of apoA1 was diluted in 1X PBS (pH 7.4) to 1/3rd of the sample volume. ApoA1 was added to the formulations by rapid T‐junction mixing at a flow rate ratio of 1: (apoA1/sample). After apoA1 addition, samples were incubated at room temperature for 20 min. Formulations were filtered through a 0.2 µm syringe filter and concentrated by centrifugal filtration in a 100 000 MWCO filter (Amicon) at 1100 G. Samples were diluted to a desired mRNA concentration and stored at 4 °C until further use.

### aNP‐mRNA Physicochemical Analysis

The hydrodynamic diameter (expressed as the number‐weighted mean), polydispersity index (PDI), and zeta potential of formulated particles were determined using a Zetasizer Nano ZSP (Malvern Instruments). For size measurements, samples were diluted 6 times in 1X PBS (pH 7.4) and equilibrated at room temperature before the measurement. Each sample was measured 3 times with 10 runs of 10 s, without fixing the attenuator and measurement position. To determine the zeta potential, formulations were diluted 20X in milliQ water and equilibrated at room temperature before measurement. Each sample was measured 5 times at 40 V for 40 runs, with a 300 s break between measurements. The efficiency of mRNA encapsulation was determined using the Quant‐it RiboGreen RNA Assay Kit (Thermo Fisher). A standard curve was generated from a dilution series of the corresponding mRNA stock. Samples were diluted in 1X Tris‐EDTA (TE) buffer and TE buffer supplemented with 2% Triton X‐100 to a theoretical mRNA concentration of 0.67 µg mL^−1^ in a black 96‐well plate to a volume of 100 µL. Next, RiboGreen reagent was diluted 200 times in TE or TE buffer containing 2% Triton X‐100. 100 µL of reagent solution was added to each well to bring the total volume to 200 µL. Fluorescence was measured on a Tecan Spark microplate reader, set at 480 nm excitation and 520 nm emission wavelengths. RNA recovery of the formulations was calculated as (amount of RNA in sample (retained + unretained))/(total amount of RNA used during formulation) x 100%. The RNA entrapment was calculated as (1‐(amount of RNA (retained+unretained))/(total amount of RNA (used in the formulation))) x 100%. The RNA retention = recovery x entrapment.

### Cryogenic Transmission Electron Microscopy (Cryo‐TEM) of aNP‐mRNA

The surface of 200‐mesh lacey carbon supported copper grids (Electron Microscopy Sciences) was exposed to plasma (40 s) using a Cressington 208 carbon coater just before vitrification. Next, 3 µL of aNP‐mRNA sample was applied on a grid and vitrified into a thin film by plunge vitrification in liquid ethane using an automated robot (FEI Vitrobot Mark IV). Cryo‐EM imaging was performed on a cryoTITAN (Thermo Fisher Scientific) with a field emission gun, a post column Gatan imaging filter (model 2002), and a post‐GIF 2k × 2k Gatan CCD camera (model 794). Micrographs were acquired at either 6500× (electron dose of 1.64 electrons A^−2^ s^−1^) or 24 000× magnification (electron dose of 11.8 A^−2^ s^−1^), at 300 kV acceleration voltage in bright‐field TEM mode with zero‐loss energy filtering (1 s acquisition time).

### In vitro EGFP‐mRNA Expression

RAW264.7 cells and primary mouse bone marrow‐derived macrophages were seeded at a density of 35000 cells/well in a 96‐well plate. The cells were left to attach overnight. Next, cells were transfected with aNPs or LNPs containing EGFP mRNA (RiboPro) at a concentration range of 0–100 ng/well. After 24 h, cells were harvested and prepared for flow cytometry by washing and staining with DRAQ7 (Thermo Fisher). Finally, cells were resuspended in flow buffer (1X PBS + 0.5% BSA + 2 mM EDTA). Data were acquired using a BD FACSAriaTM III Cell Sorter (BD Biosciences), EGFP (FIT‐C), and Alexa Fluor 700‐A (DRAQ7).

### Intravenous Injections and Cytokine Measurements

aNPs or LNPs containing mCherry mRNA (OZ Biosciences) were intravenously administered via tail vein injection to female C57BL/6J mice at doses of 0.05, 0.15, or 0.5 mg kg^−1^. After 2 h, serum was collected, and cytokine levels were measured using ELISA MAX Deluxe Set Mouse IL‐6 (BioLegend) and ELISA MAX Deluxe Set Mouse TNF‐α (BioLegend) according to the manufacturer's protocol.

### Preparing Single‐Cell Suspensions for Flow Cytometry in an In Vivo Study

Following intravenous injections with aNPs or LNPs containing mCherry mRNA (OZ Biosciences), whole blood and bone marrow were harvested from euthanized female C57BL/6J mice. Single‐cell suspensions of bone marrow were created by flushing the femurs with 10 mL flow buffer (1X DPBS + 0.5% BSA + 2 mM EDTA). Next, the bone marrow was filtered through a 70 µm cell strainer and centrifuged at 400 xg for 5 min at 4 °C. Cell pellets from blood and bone marrow were treated with 1x Red blood cell lysis (cat#420302, BioLegend). Cells were incubated for 3 × 7 min on ice (blood) or 1 min at room temperature (bone marrow). Subsequently, samples were neutralized with flow buffer and centrifuged at 400 xg for 5 min at 4 °C. Single cells were transferred to a V‐bottom 96‐well plate (Corning) and incubated with 50 µL ViaKrome 808 Fixable Viability Dye (#C36628, Beckman Coulter) diluted in 1X PBS for 20 min at room temperature, protected from light. Cells were washed, centrifuged again at 400 xg for 5 min at 4 °C, and stained with 50 µL CD16/32 (Fc‐block, 101302, BioLegend) antibodies (except for progenitor group) for 10 min on ice, protected from light. Next, cells were washed, centrifuged at 400 xg for 5 min at 4 °C, and subjected to 50 µL antibody mixture (Tables S5 and S6, Supporting Information) in brilliant stain buffer (Invitrogen) for 30 min on ice, protected from light. All data were acquired using a 21‐color CytoFLEX LX (Beckman Coulter).

### Radiolabeling of G1Ie‐aNP‐mRNA

For ^89^Zr‐ApoA1‐G1Ie‐aNP‐mRNA, apoA1 (1.1 mg mL^−1^ in PBS, pH 8.8) was combined with DFO‐p‐NCS (5 mg mL^−1^ in DMSO) and incubated for 1 h at 37 °C while stirring (600 rpm). The obtained DFO‐labeled apoA1 was directly used to formulate *aNP_8_*‐mCherry‐mRNA. For ^89^Zr‐DFO‐DPPE‐G1Ie‐aNP‐mRNA, 0.5 mol% of DMPC was exchanged for DFO‐DPPE in aNP_8_‐mCherry‐mRNA. Next, a ^89^Zr solution in 1 m oxalic acid solution was neutralized using a 2 m sodium carbonate solution until a pH of 7.2 was reached. The neutralized ^89^Zr solution was added to the DFO‐ApoA1‐aNP‐mRNA or DFO‐DPPE‐G1Ie‐aNP‐mRNA solution and incubated at 37 °C using a thermomixer (300 rpm) for 30 min. Completion of the reaction was confirmed, and a radiochemical yield above 75% was achieved, assessed by radio‐TLC (Typhoon 700IP plate reader, GE Healthcare). Samples were purified by centrifugation through Zebra 40 kDa molecular‐weight‐cutoff desalting columns to remove residual oxalic acid and unbound ^89^Zr.

### Biodistribution

According to methods developed by Perez‐Medina et al.,^[^ ^51^ ^] 89^Zr‐DFO‐DPPE‐G1Ie‐aNP‐mRNA or ^89^Zr‐ApoA1‐G1Ie‐aNP‐mRNA (0.5 mg mg^−1^ mRNA corresponding to 5–20 µCi, in 150–200 µL PBS) were intravenously administered to female C57BL/6J mice via tail vein injection. After 24 h, mice were euthanized, perfused with PBS, and tissues (blood, spleen, liver, lungs, bone, muscle, tail, bone marrow, kidneys, heart, and lymph nodes) were harvested and weighed. Next, the emitted ɣ radiation was measured by a gamma counter (2480 WIZARD2 Automatic Gamma Counter, PerkinElmer). Radioactivity values were corrected for decay and normalized to tissue weight to express radioactivity concentration as %ID per gram.

### Clinical Chemistry and Cytokine Measurements

aNP‐mRNA (0.15 mg kg^−1^) or PBS was intravenously administered to female C57BL/6J mice (n = 5 per group). After 24 h, whole blood was collected in BD Vacutainer Heparin Tubes (Becton Dickinson), and the serum was separated. Liver function (ASAT and ALAT) and renal function (urea and creatinine) were measured at the Radboudumc Laboratorium voor Diagnostiek core facility.

### Liver Section Histology

Mice were euthanized and perfused with PBS. The median liver lobes were collected and fixed in 10% formalin for 24 h. The tissues were processed and embedded in paraffin at the diagnostic pathology department at the Radboudumc. Sections of 5 µm thickness were cut using a Leica RM2235 microtome, mounted on Superfrost Plus Microscope slides (VWR), and stained with hematoxylin and eosin (H&E). The slides were scanned and visualized using CaseViewer software (3DHISTECH).

### Animals

All animal experiments conducted were approved by the Radboud University Medical Center's Dierexperimentencommissie (DEC) (CCD: AVD10300 2021 15550) and complied with both European and Dutch guidelines according to the care and use of laboratory mice. 6–8‐week‐old female C57BL/6J mice were purchased from Charles River Germany and co‐housed at ambient temperature (22–24 °C) and 45–65% RV humidity. Mice were randomized and assigned to control and treatment groups. Studies were conducted in a blinded manner.

### Statistical Analysis

All data values were expressed as mean ± SD. Data were analyzed using GraphPad Prism 10.0 by one‐way analysis of variance (ANOVA) with Dunnett's post hoc test. The difference was significant if p < 0.05. Levels of significance were indicated as follows: ^*^ *p* < 0.05; ^**^ *p* < 0.01; ^***^ *p* < 0.001; ^****^ *p* < 0.0001.

## Conflict of Interest

W.J.M.M. is a scientific co‐founder of and has equity in Trained Therapeutix Discovery and Biotrip. W.J.M.M. is CSO of Trained Therapeutix Discovery. S.R.J.H., T.A., R.Z., H.M.J., P.M.F., B.F.M.d,W., E.W.M., E.K., W.J.M.M., H.M.J, and R.v.d.M. are listed as inventors on patent applications related to this manuscript. The remaining authors declare no competing interests.

## Supporting information



Supporting Information

## Data Availability

The data that support the findings of this study are available from the corresponding author upon reasonable request.

## References

[1] K. Karikó , M. Buckstein , H. Ni , D. Weissman , Immunity 2005, 23, 165. 16111635 10.1016/j.immuni.2005.06.008

[2] J. A. Kulkarni , D. Witzigmann , S. B. Thomson , S. Chen , B. R. Leavitt , P. R. Cullis , R. van der Meel , Nat. Nanotechnol. 2021, 16, 630. 34059811 10.1038/s41565-021-00898-0

[3] D. Adams , A. Gonzalez‐Duarte , W. D. O'Riordan , C. C. Yang , M. Ueda , A. V. Kristen , I. Tournev , H. H. Schmidt , T. Coelho , J. L. Berk , K. P. Lin , G. Vita , S. Attarian , V. Planté‐Bordeneuve , M. M. Mezei , J. M. Campistol , J. Buades , T. H. 3rd Brannagan , B. J. Kim , Oh J , Y. Parman , Y. Sekijima , P. N. Hawkins , S. D. Solomon , M. Polydefkis , P. J. Dyck , P. J. Gandhi , S. Goyal , J. Chen , A. L. Strahs , et al., N. Engl. J. Med. 2018, 379, 11. 29972753 10.1056/NEJMoa1716153

[4] A. Akinc , M. A. Maier , M. Manoharan , K. Fitzgerald , M. Jayaraman , S. Barros , S. Ansell , X. Du , M. J. Hope , T. D. Madden , B. L. Mui , S. C. Semple , Y. K. Tam , M. Ciufolini , D. Witzigmann , J. A. Kulkarni , R. van der Meel , P. R. Cullis , Nat. Nanotechnol. 2019, 14, 1084. 31802031 10.1038/s41565-019-0591-y

[5] F. P. Polack , S. J. Thomas , N. Kitchin , J. Absalon , A. Gurtman , S. Lockhart , J. L. Perez , G. Pérez Marc , E. D. Moreira , C. Zerbini , R. Bailey , K. A. Swanson , S. Roychoudhury , K. Koury , P. Li , W. V. Kalina , D. Cooper , R. W. Frenck , L. L. Hammitt , Ö. Türeci , H. Nell , A. Schaefer , S. Ünal , D. B. Tresnan , S. Mather , P. R. Dormitzer , U. Sahin , K. U. Jansen , W. C. Gruber , N. Engl. J. Med. 2020, 383, 2603. 33301246 10.1056/NEJMoa2034577 PMC7745181

[6] L. R. Baden , H. M. El Sahly , B. Essink , K. Kotloff , S. Frey , R. Novak , D. Diemert , S. A. Spector , N. Rouphael , C. B. Creech , J. McGettigan , S. Khetan , N. Segall , J. Solis , A. Brosz , C. Fierro , H. Schwartz , K. Neuzil , L. Corey , P. Gilbert , H. Janes , D. Follmann , M. Marovich , J. Mascola , L. Polakowski , J. Ledgerwood , B. S. Graham , H. Bennett , R. Pajon , C. Knightly , et al., N. Engl. J. Med. 2021, 384, 403.33378609

[7] J. D. Gillmore , E. Gane , J. Taubel , J. Kao , M. Fontana , M. L. Maitland , J. Seitzer , D. O'Connell , K. R. Walsh , K. Wood , J. Phillips , Y. Xu , A. Amaral , A. P. Boyd , J. E. Cehelsky , M. D. McKee , A. Schiermeier , O. Harari , A. Murphy , C. A. Kyratsous , B. Zambrowicz , R. Soltys , D. E. Gutstein , J. Leonard , L. Sepp‐Lorenzino , D Lebwohl , N. Engl. J. Med. 2021, 385, 493. 34215024 10.1056/NEJMoa2107454

[8] H. J. Longhurst , K. Lindsay , R. S. Petersen , L. M. Fijen , P. Gurugama , D. Maag , J. S. Butler , M. Y. Shah , A. Golden , Y. Xu , C. Boiselle , J. D. Vogel , A. M. Abdelhady , M. L. Maitland , M. D. McKee , J. Seitzer , B. W. Han , S. Soukamneuth , J. Leonard , L. Sepp‐Lorenzino , E. D. Clark , D. Lebwohl , D. M Cohn , N. Engl. J. Med. 2024, 390, 432. 38294975 10.1056/NEJMoa2309149

[9] K. Musunuru , S. A. Grandinette , X. Wang , T. R. Hudson , K. Briseno , A. M. Berry , J. L. Hacker , A. Hsu , R. A. Silverstein , L. T. Hille , A. N. Ogul , N. A. Robinson‐Garvin , J. C. Small , S. McCague , S. M. Burke , C. M. Wright , S. Bick , V. Indurthi , S. Sharma , M. Jepperson , C. A. Vakulskas , M. Collingwood , K. Keogh , A. Jacobi , M. Sturgeon , C. Brommel , E. Schmaljohn , G. Kurgan , T. Osborne , H. Zhang , et al., N. Engl. J. Med. 2025, 392, 2235. 40373211 10.1056/NEJMoa2504747 PMC12713542

[10] N. Pardi , F. Krammer , Nat Rev Drug Discov 2024, 23, 838. 39367276 10.1038/s41573-024-01042-y PMC13101425

[11] A. Akinc , W. Querbes , S. De , J. Qin , M. Frank‐Kamenetsky , K. N. Jayaprakash , M. Jayaraman , K. G. Rajeev , W. L. Cantley , J. R. Dorkin , J. S. Butler , L. Qin , T. Racie , A. Sprague , E. Fava , A. Zeigerer , M. J. Hope , M. Zerial , D. W. Sah , K. Fitzgerald , M. A. Tracy , M. Manoharan , V. Koteliansky , A. D. Fougerolles , M. A. Maier , Mol. Ther. 2010, 18, 1357. 20461061 10.1038/mt.2010.85 PMC2911264

[12] D. Witzigmann , J. A. Kulkarni , J. Leung , S. Chen , P. R. Cullis , R. van der Meel , Adv Drug Deliv Rev 2020, 159, 344. 32622021 10.1016/j.addr.2020.06.026 PMC7329694

[13] Q. Cheng , T. Wei , L. Farbiak , L. T. Johnson , S. A. Dilliard , D. J. Siegwart , Nat. Nanotechnol. 2020, 15, 313. 32251383 10.1038/s41565-020-0669-6 PMC7735425

[14] S. A. Dilliard , Q. Cheng , D. J. Siegwart , Proc Natl Acad Sci U S A 2021, 118, 2109256118. 10.1073/pnas.2109256118 PMC8719871 34933999

[15] S. Liu , Q. Cheng , T. Wei , X. Yu , L. T. Johnson , L. Farbiak , D. J. Siegwart , Nat. Mater. 2021, 20, 701. 33542471 10.1038/s41563-020-00886-0 PMC8188687

[16] L. Xue , G. Zhao , N. Gong , X. Han , S. J. Shepherd , X. Xiong , Z. Xiao , R. Palanki , J. Xu , K. L. Swingle , C. C. Warzecha , R. El‐Mayta , V. Chowdhary , I. Yoon , J. Xu , J. Cui , Y. Shi , M.‐G. Alameh , K. Wang , L. Wang , D. J. Pochan , D. Weissman , A. E. Vaughan , J. M. Wilson , M. J. Mitchell , Nat. Nanotechnol. 2025, 20, 132. 39354147 10.1038/s41565-024-01747-6 PMC12207990

[17] C. D. Sago , M. P. Lokugamage , K. Paunovska , D. A. Vanover , C. M. Monaco , N. N. Shah , M. Gamboa Castro , S. E. Anderson , T. G. Rudoltz , G. N. Lando , P. Munnilal Tiwari , J. L. Kirschman , N. Willett , Y. C. Jang , P. J. Santangelo , A. V. Bryksin , J. E. Dahlman , Proc. Natl. Acad. Sci. U S A 2018, 115, E9944. 30275336 10.1073/pnas.1811276115 PMC6196543

[18] H. Kim , R. Zenhausern , K. Gentry , L. Lian , S. G. Huayamares , A. Radmand , D. Loughrey , A. R. Podilapu , M. Z. C. Hatit , H. Ni , A. Li , A. Shajii , H. E. Peck , K. Han , X. Hua , S. Jia , M. Martinez , C. Lee , P. J. Santangelo , A. Tarantal , J. E. Dahlman , Nat. Biotechnol. 2024, 10.1038/s41587-024-02470-2. PMC12095617 39578569

[19] N. Veiga , M. Goldsmith , Y. Granot , D. Rosenblum , N. Dammes , R. Kedmi , S. Ramishetti , D. Peer , Nat. Commun. 2018, 9, 4493. 30374059 10.1038/s41467-018-06936-1 PMC6206083

[20] I. Tombácz , D. Laczkó , H. Shahnawaz , H. Muramatsu , A. Natesan , A. Yadegari , T. E. Papp , M.‐G. Alameh , V. Shuvaev , B. L. Mui , Y. K. Tam , V. Muzykantov , N. Pardi , D. Weissman , H. Parhiz , Mol. Ther. 2021, 29, 3293. 34091054 10.1016/j.ymthe.2021.06.004 PMC8571164

[21] J. G. Rurik , I. Tombácz , A. Yadegari , P. O. Méndez Fernández , S. V. Shewale , L. Li , T. Kimura , O. Y. Soliman , T. E. Papp , Y. K. Tam , B. L. Mui , S. M. Albelda , E. Puré , C. H. June , H. Aghajanian , D. Weissman , H. Parhiz , J. A. Epstein , Science 1979, 375, 91. 10.1126/science.abm0594 PMC9983611 34990237

[22] T. L. Hunter , Y. Bao , Y. Zhang , D. Matsuda , R. Riener , A. Wang , J. J. Li , F. Soldevila , D. S. H. Chu , D. P. Nguyen , Q.‐C. Yong , B. Ross , M. Nguyen , J. Vestal , S. Roberts , D. Galvan , J. B. Vega , D. Jhung , M. Butcher , J. Nguyen , S. Zhang , C. Fernandez , J. Chen , C. Herrera , Y. Kuo , E. M. Pica , G. Mondal , A. L. Mammen , J. Scholler , S. P. Tanis , et al., Science 2025, 388, 1311. 40536974 10.1126/science.ads8473

[23] D. Shi , S. Toyonaga , D. G. Anderson , Nano Lett. 2023, 23, 2938. 36988645 10.1021/acs.nanolett.3c00304 PMC10103292

[24] L. Breda , T. E. Papp , M. P. Triebwasser , A. Yadegari , M. T. Fedorky , N. Tanaka , O. Abdulmalik , G. Pavani , Y. Wang , S. A. Grupp , S. T. Chou , H. Ni , B. L. Mui , Y. K. Tam , D. Weissman , S. Rivella , H. Parhiz , Science 1979, 381, 436. 10.1126/science.ade6967 PMC10567133 37499029

[25] S. R. J. Hofstraat , T. Anbergen , R. Zwolsman , J. Deckers , Y. van Elsas , M. M. Trines , I. Versteeg , D. Hoorn , G. W. B. Ros , B. M. Bartelet , M. M. A. Hendrikx , Y. B. Darwish , T. Kleuskens , F. Borges , R. J. F. Maas , L. M. Verhalle , W. Tielemans , P. Vader , O. G. de Jong , T. Tabaglio , D. K. B. Wee , A. J. P. Teunissen , E. Brechbühl , H. M. Janssen , P. M. Fransen , A. de Dreu , D. P. Schrijver , B. Priem , Y. C. Toner , T. J. Beldman , et al., Nat. Nanotechnol. 2025, 20, 532. 39900620 10.1038/s41565-024-01847-3 PMC12014499

[26] R. Huang , R. A. G. D. Silva , W. G. Jerome , A. Kontush , M. J. Chapman , L. K. Curtiss , T. J. Hodges , W. S. Davidson , Nat. Struct. Mol. Biol. 2011, 18, 416. 21399642 10.1038/nsmb.2028 PMC3079355

[27] D. P. Schrijver , A. de Dreu , S. R. J. Hofstraat , E. Kluza , R. Zwolsman , J. Deckers , T. Anbergen , K. de Bruin , M. M. Trines , E. G. Nugraha , F. Ummels , R. J. Röring , T. J. Beldman , A. J. P. Teunissen , Z. A. Fayad , R. van der Meel , W. J. M. Mulder , Adv Ther (Weinh) 2021, 4, 2100083.

[28] K. C. Vickers , B. T. Palmisano , B. M. Shoucri , R. D. Shamburek , A. T. Remaley , Nat. Cell Biol. 2011, 13, 423. 21423178 10.1038/ncb2210 PMC3074610

[29] S. C. Semple , A. Akinc , J. Chen , A. P. Sandhu , B. L. Mui , C. K. Cho , D. W. Y. Sah , D. Stebbing , E. J. Crosley , E. Yaworski , I. M. Hafez , J. R. Dorkin , J. Qin , K. Lam , K. G. Rajeev , K. F. Wong , L. B. Jeffs , L. Nechev , M. L. Eisenhardt , M. Jayaraman , M. Kazem , M. A. Maier , M. Srinivasulu , M. J. Weinstein , Q. Chen , R. Alvarez , S. A. Barros , S. De , S. K. Klimuk , T. Borland , et al., Nat. Biotechnol. 2010, 28, 172. 20081866 10.1038/nbt.1602

[30] M. Jayaraman , S. M. Ansell , B. L. Mui , Y. K. Tam , J. Chen , X. Du , D. Butler , L. Eltepu , S. Matsuda , J. K. Narayanannair , K. G. Rajeev , I. M. Hafez , A. Akinc , M. A. Maier , M. A. Tracy , P. R. Cullis , T. D. Madden , M. Manoharan , M. J. Hope , Angew. Chem. Int. Ed. Engl. 2012, 51, 8529. 22782619 10.1002/anie.201203263 PMC3470698

[31] L. B. Jeffs , L. R. Palmer , E. G. Ambegia , C. Giesbrecht , S. Ewanick , I. MacLachlan , Pharm. Res. 2005, 22, 362. 15835741 10.1007/s11095-004-1873-z

[32] L. Zhang , K. R. More , A. Ojha , C. B. Jackson , B. D. Quinlan , H. Li , W. He , M. Farzan , N. Pardi , H. Choe , NPJ Vaccines 2023, 8, 156. 37821446 10.1038/s41541-023-00751-6 PMC10567765

[33] J. A. Kulkarni , P. R. Cullis , R. van der Meel , Nucleic Acid Ther. 2018, 28, 146. 29683383 10.1089/nat.2018.0721

[34] X. Han , H. Zhang , K. Butowska , K. L. Swingle , M.‐G. Alameh , D. Weissman , M. J. Mitchell , Nat. Commun. 2021, 12, 7233. 34903741 10.1038/s41467-021-27493-0 PMC8668901

[35] K. A. Whitehead , J. R. Dorkin , A. J. Vegas , P. H. Chang , O. Veiseh , J. Matthews , O. S. Fenton , Y. Zhang , K. T. Olejnik , V. Yesilyurt , D. Chen , S. Barros , B. Klebanov , T. Novobrantseva , R. Langer , D. G. Anderson , Nat. Commun. 2014, 5, 4277. 24969323 10.1038/ncomms5277 PMC4111939

[36] X. Han , J. Xu , Y. Xu , M.‐G. Alameh , L. Xue , N. Gong , R. El‐Mayta , R. Palanki , C. C. Warzecha , G. Zhao , A. E. Vaughan , J. M. Wilson , D. Weissman , M. J. Mitchell , Nat. Commun. 2024, 15, 1762. 38409275 10.1038/s41467-024-45537-z PMC10897129

[37] K. Zhou , L. H. Nguyen , J. B. Miller , Y. Yan , P. Kos , H. Xiong , L. Li , J. Hao , J. T. Minnig , H. Zhu , D. J. Siegwart , Proc. Natl. Acad. Sci. U S A 2016, 113, 520. 26729861 10.1073/pnas.1520756113 PMC4725465

[38] L. Farbiak , Q. Cheng , T. Wei , E. Álvarez‐Benedicto , L. T. Johnson , S. Lee , D. J. Siegwart , Adv. Mater. 2021, 33, 2006619. 10.1002/adma.202006619 PMC10041668 34137093

[39] F. Tack , A. Bakker , S. Maes , N. Dekeyser , M. Bruining , C. Elissen‐Roman , M. Janicot , H. M. Janssen , B. F. M. De Waal , P. M. Fransen , X. Lou , E. W. Meijer , A. Arien , M. E. Brewster , J. Controlled Release 2006, 116, 24. 10.1016/j.jconrel.2006.09.031 17718951

[40] C. DUFES , I. UCHEGBU , A. SCHATZLEIN , Adv. Drug Deliv. Rev. 2005, 57, 2177. 16310284 10.1016/j.addr.2005.09.017

[41] A. W. Bosman , H. M. Janssen , E. W. Meijer , Chem. Rev. 1999, 99, 1665. 11849007 10.1021/cr970069y

[42] D. Brabander‐van den Berg , E. W. Meijer , Angew. Chem. Int. Ed. Eng. 1993, 32, 1308.

[43] T. Akizawa , H. Origasa , C. Kameoka , Y. Kaneko , S. Kawasaki , Therap. Apheresis Dialys. 2014, 18, 122. 10.1111/1744-9987.12068 24720402

[44] K. Kono , Polym. J. 2012, 44, 531.

[45] D. A. Tomalia , Pharmaceutics. 2024, 16, 1530 39771509 10.3390/pharmaceutics16121530 PMC11676903

[46] K. J. Hassett , K. E. Benenato , E. Jacquinet , A. Lee , A. Woods , O. Yuzhakov , S. Himansu , J. Deterling , B. M. Geilich , T. Ketova , C. Mihai , A. Lynn , I. McFadyen , M. J. Moore , J. J. Senn , M. G. Stanton , Ö. Almarsson , G. Ciaramella , L. A. Brito , Mol. Ther. Nucleic Acids 2019, 15, 1. 30785039 10.1016/j.omtn.2019.01.013 PMC6383180

[47] M. J. Carrasco , S. Alishetty , M.‐G. Alameh , H. Said , L. Wright , M. Paige , O. Soliman , D. Weissman , T. E. Cleveland , A. Grishaev , M. D. Buschmann , Commun. Biol. 2021, 4, 956. 34381159 10.1038/s42003-021-02441-2 PMC8358000

[48] L. Schoenmaker , D. Witzigmann , J. A. Kulkarni , R. Verbeke , G. Kersten , W. Jiskoot , D. J. A. Crommelin , Int. J. Pharm. 2021, 601, 120586. 33839230 10.1016/j.ijpharm.2021.120586 PMC8032477

[49] M. H. Y. Cheng , J. Leung , Y. Zhang , C. Strong , G. Basha , A. Momeni , Y. Chen , E. Jan , A. Abdolahzadeh , X. Wang , J. A. Kulkarni , D. Witzigmann , P. R. Cullis , Adv. Mater. 2023, 35, 2303370. 10.1002/adma.202303370 37172950

[50] J. B. Simonsen , J. Controlled Release 2024, 373, 952. 10.1016/j.jconrel.2024.07.046 39067793

[51] C. Pérez‐Medina , E. A. Fisher , Z. A. Fayad , W. J. M. Mulder , A. J. P. Teunissen , Eur. J. Nucl. Med. Mol. Imaging 2025. 10.1007/s00259-025-07281-4 PMC12317732 40293448

[52] X. Lian , S. Chatterjee , Y. Sun , S. A. Dilliard , S. Moore , Y. Xiao , X. Bian , K. Yamada , Y.‐C. Sung , R. M. Levine , K. Mayberry , S. John , X. Liu , C. Smith , L. T. Johnson , X. Wang , C. C. Zhang , D. R. Liu , G. A. Newby , M. J. Weiss , J. S. Yen , D. J. Siegwart , Nat. Nanotechnol. 2024, 19, 1409. 38783058 10.1038/s41565-024-01680-8 PMC11757007

[53] N. Chaudhary , L. N. Kasiewicz , A. N. Newby , M. L. Arral , S. S. Yerneni , J. R. Melamed , S. T. LoPresti , K. C. Fein , D. M. Strelkova Petersen , S. Kumar , R. Purwar , K. A. Whitehead , Nat. Biomed. Eng. 2024, 8, 1483. 39363106 10.1038/s41551-024-01256-w PMC11863198

[54] E. De Lombaerde , X. Cui , Y. Chen , Z. Zhong , J. Deckers , G. Mencarelli , L. Opsomer , H. Wang , J. De Baere , S. Lienenklaus , B. N. Lambrecht , N. N. Sanders , B. G. De Geest , ACS Nano 2024, 18, 28311. 39352021 10.1021/acsnano.4c09677

[55] S.‐H. Bae , H. Choi , J. Lee , M. Kang , S. Ahn , Y. Lee , H. Choi , S. Jo , Y. Lee , H.‐J. Park , S. Lee , S. Yoon , G. Roh , S. Cho , Y. Cho , D. Ha , S.‐Y. Lee , E.‐J. Choi , A. Oh , J. Kim , S. Lee , J. Hong , N. Lee , M. Lee , J. Park , D.‐H. Jeong , K. Lee , J.‐H. Nam , Small 2024, 21, 2405618.39264000

[56] Y. Xu , S. Ma , H. Cui , J. Chen , S. Xu , F. Gong , A. Golubovic , M. Zhou , K. C. Wang , A. Varley , R. X. Z. Lu , B. Wang , B. Li , Nat. Commun. 2024, 15, 6305. 39060305 10.1038/s41467-024-50619-z PMC11282250

[57] B. Li , I. O. Raji , A. G. R. Gordon , L. Sun , T. M. Raimondo , F. A. Oladimeji , A. Y. Jiang , A. Varley , R. S. Langer , D. G. Anderson , Nat. Mater. 2024, 23, 1002. 38740955 10.1038/s41563-024-01867-3

[58] J. Witten , I. Raji , R. S. Manan , E. Beyer , S. Bartlett , Y. Tang , M. Ebadi , J. Lei , D. Nguyen , F. Oladimeji , A. Y. Jiang , E. MacDonald , Y. Hu , H. Mughal , A. Self , E. Collins , Z. Yan , J. F. Engelhardt , R. Langer , D. G. Anderson , Nat. Biotechnol. 2024, 10.1038/s41587-024-02490-y. PMC12149338 39658727

