# Supplementary material for: Dendrimers Improve Apolipoprotein Nanoparticle mRNA Delivery to Immune Cells
Source: Adv Mater. 2025 Sep 12;38(3):e04830. doi: 10.1002/adma.202504830 (PMC12801360; doi:10.1002/adma.202504830)
Supplement: Supplementary file 1 — Supporting Information [file ADMA-38-e04830-s001.docx]

Supporting Information

**Dendrimers Improve Apolipoprotein Nanoparticle mRNA Delivery To Immune Cells**

*Mirre M. Trines^#^, Daniek Hoorn^#^, Stijn R.J. Hofstraat^#^, Robby C. Zwolsman, Tom Anbergen, Iris Versteeg, Yuri van Elsas, Jeroen Deckers, Merel M.A. Hendrikx, Teun Kleuskens, Youssef B. Darwish, Gijs W.B. Ros, Sjoerd F. Dijkstra, Bram Priem, Matt Timmers, P. Michel Fransen, Maarten J. Pouderoijen, Bas F.M. de Waal, E. W. Meijer, Thijs J. Beldman, Yohana C. Toner, Ewelina Kluza, Willem J.M. Mulder, Henk M. Janssen^*†^, Roy van der Meel^*^*

M.M. Trines, D. Hoorn, S.R.J. Hofstraat, R.C. Zwolsman, M.M.A. Hendrikx, T. Kleuskens, Y.B. Darwish S.F. Dijkstra, M. Timmers, E. Kluza, W.J.M. Mulder, R. van der Meel

Laboratory of Chemical Biology

Department of Biomedical Engineering and Institute for Complex Molecular Systems (ICMS)

Eindhoven University of Technology

Eindhoven, 5600 MB, The Netherlands

E-mail: [r.v.d.meel@tue.nl](mailto:r.v.d.meel@tue.nl)

T. Anbergen, I. Versteeg, Y. Van Elsas, J. Deckers, G.W.B. Ros, B. Priem, T.J. Beldman, Y.C Toner, W.J.M. Mulder

Department of Internal Medicine and Radboud Center for Infectious Diseases (RCI)

Radboud University Medical Center

Nijmegen, 6525 GA, The Netherlands

M.M.A Hendrikx

Biotrip B.V.

Eindhoven, 5641 AM, The Netherlands

P.M. Fransen, M.J. Pouderoijen, H.M. Janssen^†^

SyMO-Chem B.V.

Eindhoven, 5612 AZ, The Netherlands

B.F.M. De Waal, E.W. Meijer

Institute for Complex Molecular Systems

Department of Chemical Engineering and Chemistry

Eindhoven University of Technology

Eindhoven, 5600 MB, The Netherlands

^#^ M.M.T., D.H., and S.R.J.H. contributed equally to this work.

^*^ H.M.J. and R.v.d.M. contributed equally to this work.

† This publication is dedicated to Henk M. Janssen, who was instrumental for the development of the polyvalent ionizable cationic dendrimers described in this study and unfortunately passed away before its completion. He was an extraordinary human being, a beloved colleague, and a brilliant chemist who will be dearly missed.

**Experimental**

**Materials**

All reagents, chemicals, materials and solvents were obtained from commercial sources and were used as received. All solvents were AR quality and obtained from Biosolve. The PPI dendrimers were obtained from SyMO-Chem B.V.. PAMAM dendrimers were obtained from Merck as a 20 wt. % solution in methanol. The epoxides and acrylates (stabilized with MeHQ) were obtained from TCI Europe.

**Instrumentation**

^1^H-NMR and ^13^C-NMR spectra were recorded on a Bruker Avance III HD (400 MHz for ^1^H-NMR and 100 MHz for ^13^C-NMR) spectrometer at 25°C. Chemical shifts are reported in ppm downfield from TMS at 25°C. Abbreviations used for splitting patterns are s=singlet, t=triplet, q=quartet, m=multiplet and br=broad. HPLC-MS/PDA was performed using a Shimadzu LC-20 AD VP series HPLC coupled to a diode array detector (Shimadzu SPD-M20A) and an Ion-Trap (LCQ Fleet, Thermo Scientific) MS-detector, employing a Phenomenex Kinetex 5μm EVO C_18_ 100 Å column using an injection volume of 1-10 μL, a flow rate of 0.3 mL/mL and typically a gradient (5% to 100% in 5 min, held at 100% for 1 min) of acetonitrile in H_2_O (both containing 0.1 v/v% formic acid) at 20°C. The MALDI TOF MS measurements were performed with an Autoflex Speed (Bruker, Bremen, Germany) instrument. α-Cyano-4-hydroxycinnamic acid (CHCA)was used as the matrix. The accelerating voltage was held at 19 kV and the delay time at 130 ns. Mass spectra were acquired in the reflector positive ion mode by summing spectra from 500 random laser shots at an acquisition rate of 100 Hz.

**Dendrimer synthesis**

*Synthesis of G2Ie*

This synthesis describes the Michael-addition reaction of G2-PPI-(NH_2_)_8_ dendrimer with *n*-octyl acrylate, leading to fully functionalized PPI-dendrimer with 16 octyl*-*ester groups.


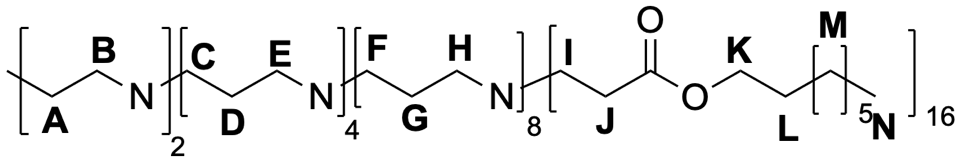


G2 PPI dendrimer (*n*-butylene core; 0.20 g; 0.258 mmol; 2.06 mmol primary amine groups) was dissolved in iso-propanol (1 mL). An excess of *n*-octyl acrylate (1.73 mL, 1.53 g, 8.27 mmol; 32 mol eqs) was added and the reaction mixture was stirred in a closed vial at 55°C under an inert atmosphere of nitrogen. The progress of the reaction was monitored with ^1^H-NMR. The mixture was stirred for 4 days, after which conversion was complete. The mixture was evaporated in vacuo to dryness, and the residue was stirred in MeCN (15 mL) at 4°C. After 3-4 hours, the supernatant was decanted (or pipetted off) to remove the excess of acrylate. Washing with MeCN was repeated two more times at 4°C, and one final time at -20°C (with longer-chain acrylates this can lead to precipitation of the acrylate). The product was dried in vacuo to yield a slightly yellowish oil. Yield: 515 mg (54%). The ^1^H-NMR spectrum was in agreement with the desired structure.^1^H-NMR (400 MHz, Chloroform-d) δ 4.04 (t, J = 6.8 Hz, 32H, K), 2.77 (t, J = 7.3 Hz, 32H, I), 2.54 – 2.27 (m, 84H, B, C, E, F, H, J), 1.58 (dt, J = 22.2, 6.9 Hz, 60H, A, D, G, L), 1.30 (dd, J = 14.0, 7.7 Hz, 160H, M), 1.08 – 0.64 (m, 48H, N).MALDI-TOF-MS (CHCA matrix, positive reflector mode): Obs. *m/z* = (M+H)^+^ 3722.19. Calculated: C_216_H_416_N_14_O_32_ (exact mass 3719.14; molecular weight 3721.77).

*Synthesis of G1Ie*


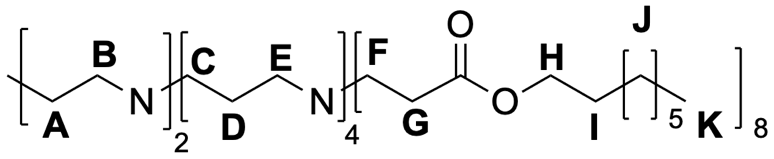


The reaction between G1-PPI-(NH_2_)_4_ (*n*-butylene core) (200 mg, 0.63 mmol) and *n*-octyl acrylate (1.86 g, 10.1 mmol, 16 eq) in iso-propanol (2 ml) was performed in a similar way as done for dendrimer G2Ie. Yield: 758 mg (67%). The ^1^H-NMR spectrum was in agreement with the desired structure.^1^H NMR (400 MHz, Chloroform-d) δ 4.05 (t, J = 6.8 Hz, 16H, H), 2.77 (t, J = 7.4 Hz, 16H, F), 2.40 (dt, J = 23.2, 7.5 Hz, 36H, B, C, E, G), 1.58 (dt, J = 23.6, 6.7 Hz, 28H, A, D, I), 1.47 – 1.07 (m, 80H, J), 1.07 – 0.57 (m, 24H, K).MALDI-TOF-MS (CHCA matrix, positive reflector mode): Obs. *m/z* = (M+H)^+^ 1791.52, (M+Na)^+^ 1813.52, (M+K)^+^ 1829.49. Calculated: C_104_H_200_N_6_O_16_ (exact mass 1789.50; molecular weight 1790.77).

*Synthesis of G1Ib*


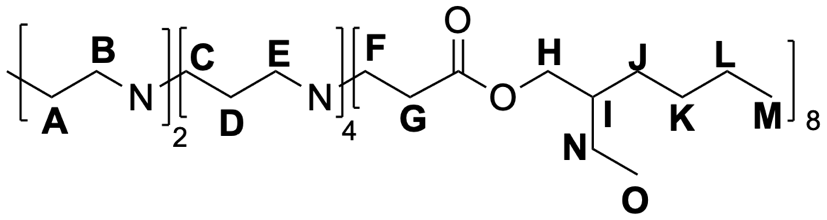


The reaction between G1-PPI (DAB-Am-4) (174 mg, 0.59 mmol) and 2-ethyl-hexyl acrylate (1.013 g, 5.5 mmol,10 eq) in iso-propanol (2 ml) was performed in a similar way as done for dendrimer G2Ie. Yield: 0.71 g (0.4 mmol, 73%). The ^1^H-NMR spectrum was in agreement with the desired structure.^1^H NMR (399 MHz, Chloroform-*d*) δ 4.36-3.57 (m, 16H, H), 2.78 (t, *J* = 7.4 Hz, 16H, F), 2.41 (dt, *J* = 25.0, 7.5 Hz, 35H, G , C, E, B), 1.69-1.48 (m, 16H), 1.48-1.11 (m, 68H), 0.89 (td, *J* = 7.1, 2.8 Hz, 48H, M,O). MALDI-TOF-MS (CHCA matrix, positive reflector mode): Obs. *m/z* = (M+H)^+^ 1791.55, (M+Na)^+^ 1813.53. Calculated: C_120_H_232_N_6_O_16_ (exact mass 1789.50; molecular weight 1790.77).

*Synthesis of G1Mb*


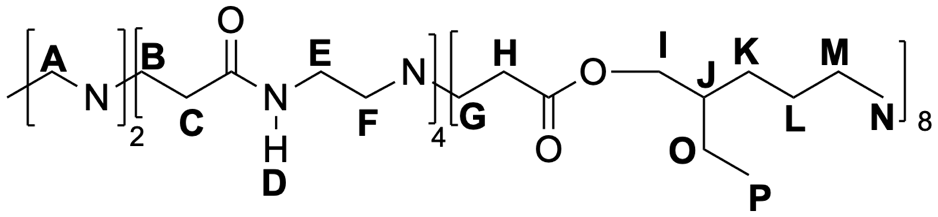


To a reaction tube with a stirred solution of G1-PAMAM-C2 (ethylene-diamine core; 4 amine end groups; 165 mg, 0.32 mmol) in propan-2-ol (IPA; 2 mL) was added 2-ethyl-hexylacrylate (589 mg, 3.2 mmol, 10 eq). More 2-ethyl-hexylacrylate was later added (600 mg, 3.3 mmol, 10 eq). The reaction mixture was heated to 60°C for 408 h. The reaction mixture was concentrated in *vacuo*. The residue was evaporated further with oil pump to remove excess of 2-ethyl-hexylacrylate (at 90°C and 0 mbar). The crude product still contained a small amount of acrylate that was further removed using a short silica column (starting 2% MeOH/CHCl_3_ to 10% MeOH/CHCl_3_) yielding a yellow viscous oil (270 mg, 0.13 mol, 41%).^1^H NMR (400 MHz, Chloroform-*d*) δ 7.20 (t, *J* = 5.5 Hz, 3H), 4.19-3.78 (m, 16H, I), 3.27 (q, *J* = 6.0 Hz, 8H, B), 2.78 (dt, *J* = 15.0, 6.9 Hz, 22H, F, G), 2.57 (t, *J* = 6.4 Hz, 9H, C), 2.52 (s, 3H, A), 2.44 (t, *J* = 7.1 Hz, 15H, H), 2.34 (t, *J* = 6.4 Hz, 7H, E), 1.63-1.47 (m, 8H, J), 1.44-1.12 (m, 64H, K, L, M, O), 0.89 (td, *J* = 7.5, 7.0, 3.0 Hz, 48H, N, P). MALDI-TOF-MS (CHCA matrix, positive reflector mode): Obs. *m/z* = (M+H)^+^ 1991.56, (M+Na)^+^ 2013.53. Calculated: C_110_H_208_N_10_O_20_ (exact mass 1989.56; molecular weight 1990.92).

*Synthesis of G1Me*


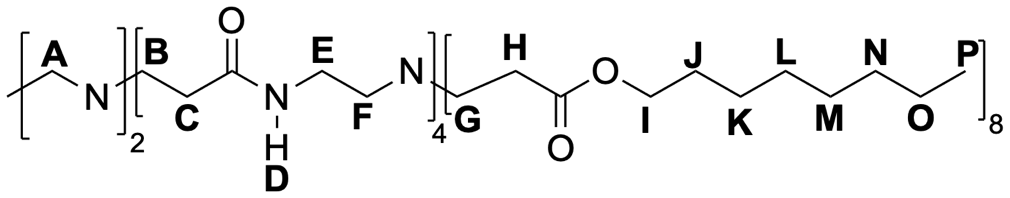


To a reaction tube with a stirred solution of G1-PAMAM-C2 (ethylene-diamine core; 4 amine end groups; 309 mg, 059 mmol) in propan-2-ol (IPA; 1 mL) was added octyl-acrylate (1.76g, 9.56 mmol, 16 eq). The reaction mixture was heated to 60°C for 240 h. The reaction mixture was concentrated in *vacuo*. The residue was stirred well in MeCN and cooled down to -20°C, allowing the product to phase separate from the MeCN-layer. The crude product still contained a small amount of acrylate that was further removed using a short silica column (starting 5% MeOH/CHCl_3_) yielding a yellow viscous oil (176 mg, 14 %). ^1^H NMR (399 MHz, Chloroform-*d*) δ 7.22 (d, *J* = 5.4 Hz, 3H), 4.05 (t, *J* = 6.8 Hz, 16H, I), 3.27 (q, *J* = 6.0 Hz, 7H, B), 2.77 (q, *J* = 8.1, 7.6 Hz, 22H, f, G), 2.55 (q, *J* = 9.1, 7.7 Hz, 13H, A, C), 2.43 (t, *J* = 7.0 Hz, 16H, H), 2.35 (s, 6H, E), 1.62 (q, *J* = 7.0 Hz, 16H, J), 1.52 – 1.08 (m, 81H, K, L, M, N, O, ), 1.08 – 0.50 (m, 25H, P).MALDI-TOF-MS (CHCA matrix, positive reflector mode): Obs. *m/z* = (M+H)^+^ 1991.56, (M+Na)^+^ 2013.53. Calculated: C_110_H_208_N_10_O_20_ (exact mass 1989.56; molecular weight 1990.92).

*Synthesis of G0e*


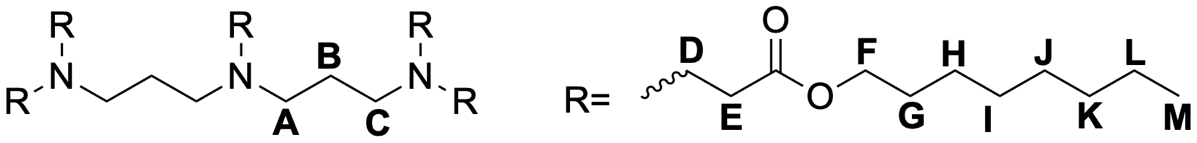


To a reaction tube with a stirred solution of bis(3-aminopropyl)amine (131 mg, 1 mmol) in IPA (1 ml) was added *n*-octyl-acrylate (1.843 g, 10 mmol, 10 eq). The reaction mixture was heated to 60°C for 136h. The reaction mixture was concentrated in *vacuo*. The residue was stirred well in MeCN and cooled down to -20°C, allowing the product to phase separate from the MeCN-layer. The supernatant was carefully removed with a pipette and the procedure was repeated five times. The residue was dried on a vacuum line yielding a clear colorless oil (769 mg, 0.731 mmol, 73%) ^1^H NMR (400 MHz, Chloroform-*d*) δ 4.05 (t, *J* = 6.8 Hz, 10H, F), 2.76 (td, *J* = 7.4, 3.8 Hz, 10H, D), 2.41 (dt, *J* = 10.7, 6.8 Hz, 18H, A, C, E), 1.74 – 1.48 (m, 14H, G), 1.42 – 1.16 (m, 50H, H, I, J, K, L), 0.95 – 0.80 (m, 15H, M). ^13^C NMR (101 MHz, CDCl_3_) δ 172.87, 172.71, 64.58, 64.55, 51.83, 51.79, 49.26, 49.20, 32.57, 32.27, 31.81, 29.26, 29.21, 28.67, 28.66, 25.94, 24.80, 22.65, 14.10. MALDI-TOF-MS (CHCA matrix, positive reflector mode): Obs. *m/z* = (M+H)^+^ 1052.85, (M+Na)^+^ 1074.84, (M+K)^+^ 1090.80. Calculated: C_61_H_117_N_3_O_10_ (exact mass 1051.87; molecular weight 1052.62).

*Synthesis of G0b*


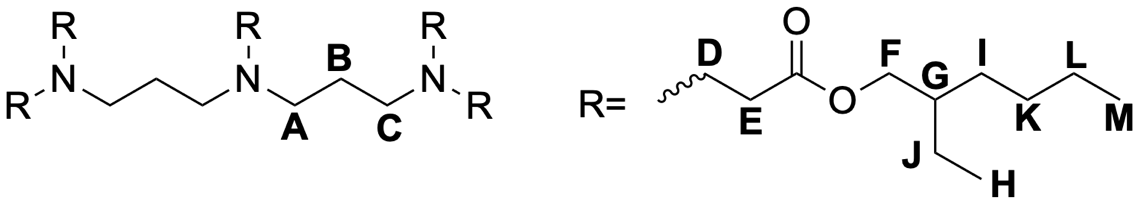


2-Ethyl-hexyl-acrylate (1.4 g, 7.5 mmol, 10 eq) was added to a stirred solution of bis(3-aminopropyl)amine (100 mg, 0.75 mmol) in iso-propanol (2 mL). Components were reacted in a similar way as described for dendrimer G2Ie. Yield after work-up: 278 mg (0.26 mmol, 35 %).^1^H NMR (399 MHz, Chloroform-*d*) δ 4.19-3.78 (m, 10H, F), 2.77 (t, *J* = 7.3 Hz, 10H, D), 2.56-2.25 (m, 17H, A, C ,E), 1.67 (s, 1H), 1.64-1.48 (m, 9H, B, G), 1.48-1.11 (m, 40H, I, J, K, L), 0.89 (td, *J* = 7.1, 2.8 Hz, 30H, M). MALDI-TOF-MS (CHCA matrix, positive reflector mode): Obs. *m/z* = (M+H)^+^ 1052.88, (M+Na)^+^ 1074.86. Calculated: C_59_H_113_N_3_O_10_ (exact mass 1051.87; molecular weight 1052.62).

*Synthesis of G1Ia*


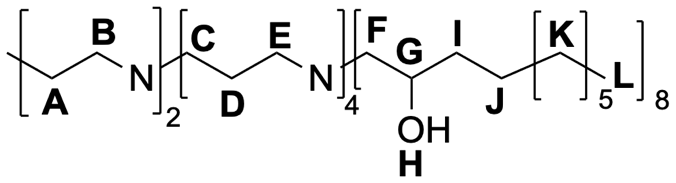


This synthesis describes the reaction of G1-PPI-(NH_2_)_4_ dendrimer with 1,2-epoxy-dodecane leading to PPI-dendrimer with 2-hydroxy C10 groups. PPI dendrimer G1 (0.10 gram 0.315 mmol) was dissolved in iso-propanol (1 mL) and 1,2-epoxy-dodecane (0.58 g, 10 mol eqs) was added. The reaction mixture was stirred in a closed vial at 90°C under an inert atmosphere of nitrogen for 24 hours and was then evaporated to dryness. The residue was stirred in MeCN and the suspension was put at -20°C for several hours. The supernatant was decanted. This procedure was repeated two times, after which the residue did not contain any remaining 1,2-epoxy-dodecane anymore. The residue was dried in vacuo, giving the product. Yield: 447 mg (79%). The ^1^H-NMR spectrum was in agreement with the desired structure. ^1^H NMR (400 MHz, CDCl_3_/CD_3_OD) δ 3.60 (pt, *J* = 6.4, 3.5 Hz, 8H G), 3.49 (s, 10H), 3.38 (t, *J* = 1.8 Hz, 1H), 2.79 – 2.16 (m, 34H, B, C, E, F), 1.61 (p, *J* = 7.0 Hz, 8H D), 1.27 (d, *J* = 6.1 Hz, 146H, A, I, J, K), 0.88 (t, *J* = 6.7 Hz, 24H, L).

MALDI-TOF-MS (CHCA matrix, positive reflector mode): Obs. m/z = (M+H)+ 1791.83, (M+Na)+ 1813.82, (M+K)+ 1829.79. Calculated: C112H232N6O8 (exact mass 1789.79; molecular weight 1791.12).

*Synthesis of G2Ia*


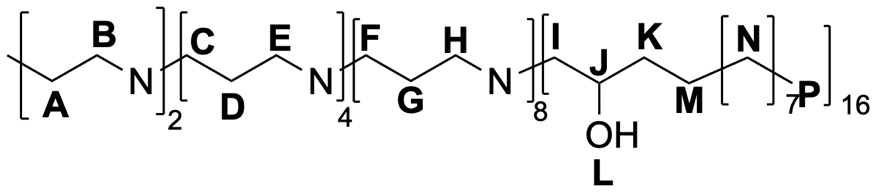


The reaction between G2-PPI-(NH_2_)_8_ (*n*-butylene core) (170 mg, 0.22 mmol) and 1,2-epoxy-dodecane (810 mg, 4.39 mmol, 20 eq) in isopropanol (3 ml) was performed in a similar way as dendrimer G1la. Yield: 697 mg (84%). The ^1^H-NMR spectrum was in agreement with the desired structure. ^1^H NMR (400 MHz, Chloroform-*d*) δ 4.22 (s, 12H, L), 3.60 (dq, *J* = 12.3, 6.4, 5.6 Hz, 16H, J), 2.76 – 2.16 (m, 82H, B, C, E, F, H,I), 1.79 – 1.52 (m, 23H, D, G), 1.26 (d, *J* = 4.1 Hz, 300H, A, K, M, N ), 0.88 (t, *J* = 6.7 Hz, 50H, O). MALDI-TOF-MS (CHCA matrix, positive reflector mode): Obs. *m/z* = (M+H)^+^ 3722.78, (M+Na)^+^ 3744.75, (M+K)^+^ 3760.74. Calculated: C_232_H_480_N_14_O_16_ (exact mass 3719.72; molecular weight 3722.47).

*Synthesis of G0a*


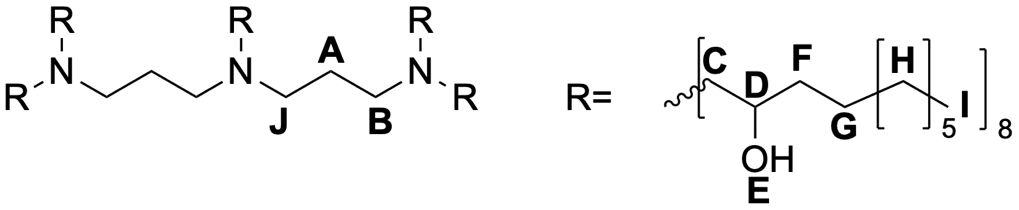


The reaction between bis(3-aminopropyl)amine (250 mg ,1.9 mmol) and 1,2-epoxydecane (1.86 g, about 11.9 mmol, 6.26 eq) in isopropanol (1 ml) was performed in a similar way as dendrimer G1la. Yield (1.56 g 90%).^1^H NMR (400 MHz, CDCl_3_/CD_3_OD) δ 3.78 – 3.48 (m, 5H, D), 2.85 – 2.16 (m, 22H,B, F, J), 1.63 (dt, *J* = 14.1, 6.3 Hz, 4H, A), 1.54 – 1.08 (m, 69H, F, G, H), 1.08 – 0.60 (m, 15H, I).MALDI-TOF-MS (ODCB matrix, positive reflector mode): Obs. *m/z* = (M+H)^+^ 912.87. Calculated: C_56_H_117_N_3_O_5_ (exact mass 911.90; molecular weight 912.57).

*Synthesis of G1Ma*


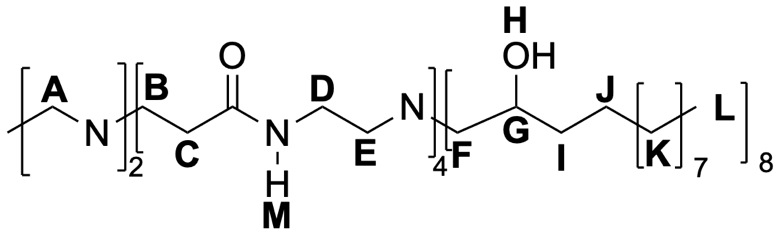


To a reaction tube with a stirred solution of G1-PAMAM-C2 (ethylene-diamine core; 4 amine end groups; 316 mg, 0.61 mmol) in propan-2-ol (IPA; 1 mL) was added 1,2-epoxy-dodecane (1.12 g, 6.1 mmol, 10 eq). The reaction mixture was heated to 60°C for 48 h. The reaction mixture was concentrated in *vacuo*. The residue was stirred in MeCN and cooled down to -20°C, allowing the product to phase separate from the MeCN solvent. The supernatant was carefully removed using a pipette, and the trituration procedure was repeated three times. The crude product still contained a small amount of impurities which were further removed using a short silica column (starting 2% MeOH/CHCl_3_ to 10% MeOH/CHCl_3_) yielding a viscous oil (0.653g, 54%). ^1^H NMR (399 MHz, Chloroform-*d*) δ 7.96 (d, *J* = 75.1 Hz, 4H, M), 4.54 (s, 7H, H), 3.60 (d, *J* = 14.5 Hz, 8H, G), 3.49 (s, 2H), 3.39 – 3.20 (m, 4H), 3.12 (s, 2H), 2.94 – 1.96 (m, 43H, A, B, C, E, F), 1.72 – 1.07 (m, 143H, I, J, K ), 0.88 (t, *J* = 6.7 Hz, 25H, L). MALDI-TOF-MS (CHCA matrix, positive reflector mode): Obs. *m/z* = (M+H)^+^ 1991.56, (M+Na)^+^ 2013.53. Calculated: C_110_H_208_N_10_O_20_ (exact mass 1989.56; molecular weight 1990.92.

**Additional experimental data**

|  |
| --- |
| **Figure S1.** pKa data of polyvalent ionizable cationic dendrimers as determined by a TNS assay. Data represents mean ± SD (n=3 experiments). |

| ** |
| --- |
| **Figure S2.** Percentage of EGFP-positive RAW264.7 cells 24 hours after in vitro transfection with different G1Ie- or G1Me-based aNP-mRNA compositions. The percentage of EGFP positive cells was determined with flow cytometry. Data represents mean ± SD (n=3 experiments). |

|  |
| --- |
| **Figure S3.** Extensive cryo-TEM overview of dendrimer-aNPs and ALC-0315 aNP as control. **Top panels.** Cryo-TEM image at a 24000-fold magnification. The scalebar represents a size of 50 nm. **Bottom panels.** Overview of the formulation at a 6500-fold magnification. The scale bar represents a size of 500 nm. |

|  |
| --- |
| **Figure S4.** Percentage of EGFP-positive cells (*top panels*) and cell viability (*bottom panels*) 24 hours after aNP-mRNA treatment in A) RAW264.7 macrophages and B) murine bone marrow-derived macrophages. Data represents mean ± SD (n=3-4 experiments) and was analyzed using a one-way ANOVA with Dunnett’s post hoc test. Significant differences between ALC-0315 (ALC)-based aNP-mRNA (100 or 200 ng) versus dendrimer-based aNP-mRNA or LNP-mRNA (100 or 200 ng) are indicated by: * indicates statistical difference (p ≤ 0.05), ** indicates statistical difference (p ≤ 0.01), *** indicates statistical difference (p ≤ 0.001), **** indicates statistical difference (p ≤ 0.0001), # indicates statistical difference (p ≤ 0.0001) from all other groups. |

|  |
| --- |
| **Figure S5.** Molecular structures of selected dendrimers for in vivo evaluation. A) G1Ib. B) G1Me. C) G1Mb. |

|  |
| --- |
| **Figure S6.** Flow cytometry gating approach for A) myeloid cells and B) progenitor cells. |

|  |
| --- |
| **Figure S7.** mCherry expression indicated by geometric mean fluorescence intensity (gMFI, left panels) and percentage (%) positive cells (right panels) in A) bone marrow monocytes, B) blood monocytes, C) hematopoietic stem cells (HSCs), and D) myeloid progenitors (MyPs) stem and progenitor cells 19 hours following i.v. injection of LNP or aNP containing mCherry mRNA at a dose of 0.05, 0.15 or 0.5 mg/kg. Data represents mean ± SD (n=3-5 mice) and was analyzed using a one-way ANOVA with Dunnett’s post hoc test. Significant differences between ALC-0315 (ALC)-based aNP-mRNA versus dendrimer-based aNP-mRNA or LNP-mRNA (dose 0.15 mg/kg) are indicated by: * indicates statistical difference (p ≤ 0.05), ** indicates statistical difference (p ≤ 0.01), *** indicates statistical difference (p ≤ 0.001). |

|   **Figure S8 continues on the next page.** |
| --- |
| **** |

| **Figure S8.** (A) Interleukin-6 (IL-6) serum levels at different doses and (B) Tumor Necrosis Factor (TNF) serum levels 2 hours after i.v. injection of dendrimer-based aNPs at a dose of 0.15 mg/kg. Data represents mean ± SD (n=3-5 mice) and was analyzed using a one-way ANOVA with Dunnett’s post hoc test. Significant differences between ALC-0315 (ALC)-based aNP-mRNA versus dendrimer-based aNP-mRNA or LNP-mRNA (dose 0.15 mg/kg) are indicated by: ** indicates statistical difference (p ≤ 0.01), *** indicates statistical difference (p ≤ 0.001). C) Biocompatibility of dendrimer mRNA-aNPs as determined by serum levels of alanine aminotransferase (ALAT), aspartate aminotransferase (ASAT), creatinine and urea 24 hours after intravenous administration in mice. Data is expressed as mean ± SD (n=3-5 mice) and analyzed using a one-way ANOVA with Dunnett’s post hoc test. D) Paraffin liver sections (5 μm thickness) stained with hematoxylin and eosin. Presented are liver sections from mice treated with PBS (n=1) or mRNA formulations (LNP, ALC, G1Ie, G1Ib, G1Me or G1Mb, n=2). Scale bar represents 100 μm. |
| --- |

| **Table S1.** aNP-mRNA and LNP-mRNA compositions in mg and mol% |
| --- |
| 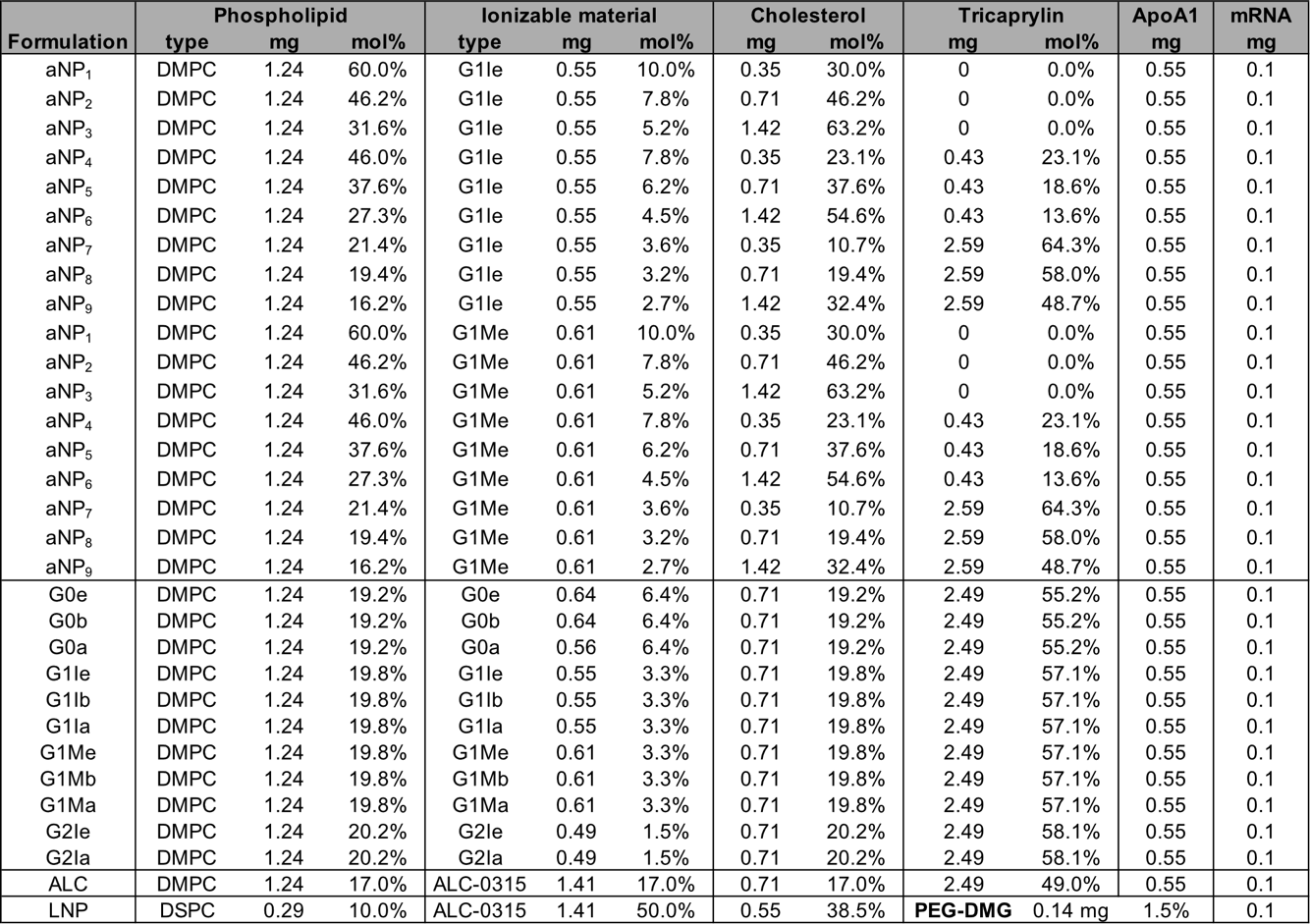 |

| **Table S2.** Numerical data of **Figure 2B**. Data represents mean ± SD (n=3 experiments). |
| --- |
| 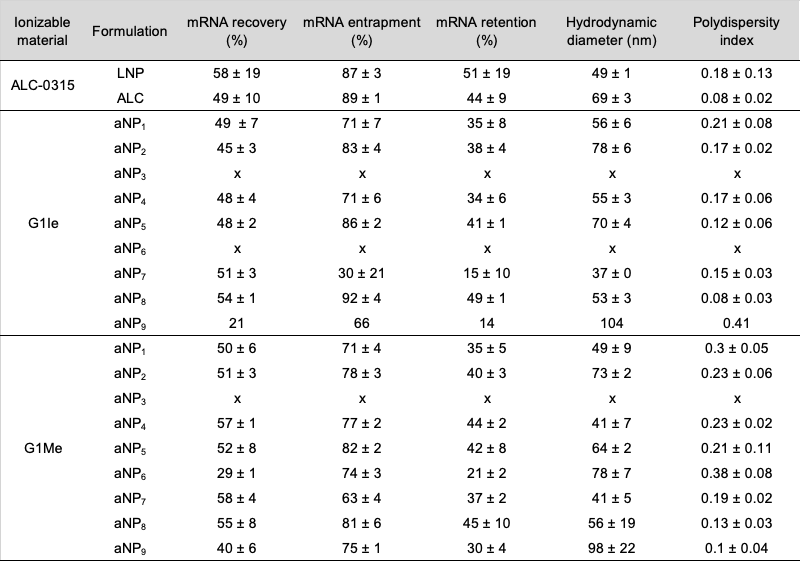 |

| **Table S3.** Numerical data of **Figure 3B**. Data represents mean ± SD (n=3 experiments). |
| --- |
| 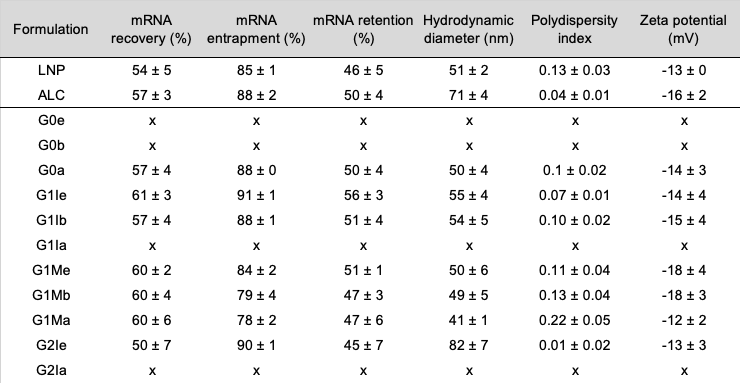 |

| **Table S4.** Physicochemical characterization of dendrimer-aNPs evaluated in vivo. Data represents mean of three technical replicates. | |
| --- | --- |
|  | |
|  |  |
|  |  |

| **Table S5.** Antibodies for myeloid cell panel. | | | | | | | | | | |
| --- | --- | --- | --- | --- | --- | --- | --- | --- | --- | --- |
| **Target** | **Fluorochrome** | **Type** | **Isotype species** | **Isotype Ig** | **Isotype fragment** | **Clone** | **Immunogen** | **Company** | **Catalog number** | **Lot/batch number** |
| CD115 (CSF-1R) | BV421 | Monoclonal | Rat | IgG2a | κ | AFS98 | - | BioLegend | 135513 | B334938 |
| F4/80 | APC | Monoclonal | Rat | IgG1 | κ | QA17A29 | Murine macrophages | BioLegend | 157306 | B336818 |
| CD11b | BV785 | Monoclonal | Rat | IgG2b | κ | M1/70 | C57BL/10 splenocytes | BioLegend | 101243 | B373879 |
| CD45 | PerCP | Monoclonal | Rat | IgG2b | κ | 30-F11 | Mouse thymus or spleen | BioLegend | 103130 | B349380 |

| **Table S6.** Antibodies for progenitor cell panel. | | | | | | | | | | |
| --- | --- | --- | --- | --- | --- | --- | --- | --- | --- | --- |
| **Target** | **Fluorochrome** | **Type** | **Isotype** | **Isotype Ig** | **Isotype fragment** | **Clone** | **Immunogen** | **Company** | **Catalog number** | **Lot/batch number** |
| CD117 (c-kit) | BV785 | Monoclonal | Rat | IgG2b | κ | ACK2 | Murine IL-3 dependent mast cells | BioLegend | 135138 | B387239 |
| Ly-6A/E (Sca-1) | APC | Monoclonal | Rat | IgG2a | κ | W18174A | EL4 Cell | BioLegend | 160904 | B342242 |
| Lineage cocktail: CD3+CD11b+CD45R/B22+Ly-76+Ly6G+Ly6C | FITC |  |  |  |  |  |  | BioLegend | 133302 |  |

**Statistical analysis**

All data values are expressed as mean ± SD. Data were analyzed using GraphPad Prism 10.0 by one-way analysis of variance (ANOVA) with Dunnett’s post hoc test. Difference was significant if p < 0.05. Levels of significance were indicated as follows: *p < 0.05; **p < 0.01; ***p < 0.001; ****p < 0.0001.
